# Computational Identification of Antibody Epitopes on the Dengue Virus NS1 Protein

**DOI:** 10.3390/molecules22040607

**Published:** 2017-04-10

**Authors:** Martina L. Jones, Fiona S. Legge, Kebaneilwe Lebani, Stephen M. Mahler, Paul R. Young, Daniel Watterson, Herbert R. Treutlein, Jun Zeng

**Affiliations:** 1 Australian Institute for Bioengineering and Nanotechnology, The University of Queensland, St. Lucia, QLD 4072, Australia; martina.jones@uq.edu.au (M.L.J.); lebanik@biust.ac.bw (K.L.); s.mahler@eng.uq.edu.au (S.M.M.); 2 ARC Training Centre for Biopharmaceutical Innovation, The University of Queensland, St. Lucia, QLD 4072, Australia; p.young@uq.edu.au; 3 Computist Bio-Nanotech, 1 Dalmore Drive, Scoresby, VIC 3179, Australia; suelegge@gmail.com; 4 School of Chemistry and Molecular Biosciences, The University of Queensland, St. Lucia, QLD 4072, Australia; d.watterson@uq.edu.au; 5 Australian Infectious Diseases Research Centre, The University of Queensland, St. Lucia, QLD 4072, Australia; 6 School of Medical Sciences, RMIT University, P.O. Box 71, Bundoora, VIC 3083, Australia

**Keywords:** antibody Epitopes, computational modeling, dengue virus

## Abstract

We have previously described a method to predict antigenic epitopes on proteins recognized by specific antibodies. Here we have applied this method to identify epitopes on the NS1 proteins of the four Dengue virus serotypes (DENV1–4) that are bound by a small panel of monoclonal antibodies 1H7.4, 1G5.3 and Gus2. Several epitope regions were predicted for these antibodies and these were found to reflect the experimentally observed reactivities. The known binding epitopes on DENV2 for the antibodies 1H7.4 and 1G5.3 were identified, revealing the reasons for the serotype specificity of 1H7.4 and 1G5.3, and the non-selectivity of Gus2. As DENV NS1 is critical for virus replication and a key vaccine candidate, epitope prediction will be valuable in designing appropriate vaccine control strategies. The ability to predict potential epitopes by computational methods significantly reduces the amount of experimental work required to screen peptide libraries for epitope mapping.

## 1. Introduction

Dengue fever is the most important human arthropod-borne viral disease with dengue virus (DENV) infections representing one of the biggest public health burdens globally. More than 400 million infections and 100 million symptomatic cases occur annually, with as many as 3.6 billion people at risk of infection in areas where *Aedes aegypti*, the major mosquito vector, is found [1]. Treatment is limited to palliative care, as currently there is no effective antiviral drug or vaccine clinically available. The recent failure of the leading candidate vaccine currently in clinical trials, to elicit solid cross-protection against all four dengue serotypes, highlights the pressing need for maintaining research efforts that could lead to additional control options.

Dengue virus has four serotypes (DENV1–4), all of which are responsible for the spectrum of disease ranging from benign dengue fever to severe DHF/DSS [2,3]. The DENV genome contains three structural proteins (capsid, pre-membrane and envelope) and seven nonstructural proteins (NS1, NS2a, NS2b, NS3, NS4a, NS4b and NS5) [4]. Amongst them, NS1 is a 46–50 kDa glycoprotein expressed in infected mammalian cells in membrane-associated (mNS1) and secreted (sNS1) forms [5]. DENV NS1 is known to be a major target of humoral immunity in DENV infection. In addition, protective immune responses can be elicited from vaccination with flavivirus NS1 [6,7] thereby identifying it as a candidate immunogen for inclusion in subunit vaccine strategies. However, some antibodies to NS1 are known to cross-react with the viral envelope protein, and are associated with lethal antibody-enhanced replication (AER) of subsequent viral infections [8]. Therefore, identifying antigenic epitopes of DENV NS1 which bind to neutralizing antibodies that don't elicit AER is an important step in both the development of effective subunit vaccines and monoclonal antibodies with therapeutic potential to treat DHF and DSS. A recent study on the domain III of dengue virus envelope protein has led to the rational design of a tetravalent dengue vaccine candidate by using several in-silico analysis tools [9].

Previously, we developed a new approach for determining linear antibody-binding epitopes of antigens [10,11]. This approach has been successfully used in the following examples; to identify important epitopes on the HIV gp120 envelope glycoprotein that are recognized by human neutralizing antibodies, to predict epitopes on a bunyavirus glycoprotein ectodomain recognition of severe fever with thrombocytopenia syndrome (SFTS) virus to its human antibody Mab 4–5 [11] and Shiga Toxin 2 (Stx2) subunit A to its specific antibodies 11E10 and S2C2 [10]. Briefly, epitope identification involves three steps. Firstly, the identification of the locations of chemical functional groups on key regions of the antibody using an exhaustive “multiple copy simultaneous search” (MCSS) approach [12,13,14,15,16,17]. Each of these functional groups corresponds to an individual amino acid [16]. Secondly, MCSS clusters of a specific functional group with favorable interaction energies with the protein, also referred to as “minima”, are selected to derive the pattern of functional groups on the surface of the antigen. These functional group patterns are subsequently converted into the amino acid sequence pattern. Thirdly, the antigen protein sequence is separated into short peptides of seven amino acids, which are scored according to the number of matched amino acids with the identified sequence pattern. The peptides with the highest scores matching the key pattern are considered to be mimotopes [10,11]. This method originated from our computational combinatorial inhibitor design (CCLD) approach [14,16,17], which has been used previously to design peptide inhibitors of Ras-Raf interactions [14,15,16]. Several of these peptide inhibitors were subsequently confirmed by in vitro Enzyme-Linked ImmunoSorbent Assay (ELISA), radioassay and biosensor-based assays [15].

Recently, Jiang et al. used a phage display technique to identify epitopes on the NS1 protein of DENV2 recognized by a polyclonal antibody [18]. Being a polyclonal antibody, several linear epitopes were identified, defined by residues 32–40, 80–89, 103–112, 121–130, 187–196, 295–304 and 315–324 [18]. Falconar et al. [19] had earlier screened 34 mouse monoclonal antibodies (mAbs), and identified linear binding epitopes on DENV2 NS1 for nine of these mAbs by screening 174 overlapping peptides. These epitopes were referred to as LD2 (residues 25–33; recognised by mAbs 5H4.4 and 1H7.4), 24A (residues 61–69; recognised by mAb 5H4.3), LX1 (residues 111–121; recognised by mAbs 3D1.4, 4H3.4, 3A5.4 and 1A12.3), and 24C (residues 299–309; recognised by mAbs 5H5.4 and 1G5.3). The four mAbs binding the LX1 epitope cross-react with all four dengue virus serotypes, reflective of the sequence homology of NS1 at this site, whilst the antibodies binding the other epitopes show some serotype selectivity. The remaining 25 mAbs are assumed to bind conformational epitopes.

More recently, the crystal structure of the DENV2 NS1 protein (PDB code: 4O6B) has been solved in both dimeric and hexameric configurations thereby revealing the molecular basis of their formation [20]. The crystal structure of the DENV2 NS1 protein also provides us with a useful guide for the selection of potential epitopes as more recent studies have shown that some of the previously predicted epitopes could be inside the core region of the antigen, and therefore potentially inaccessible [10,11].

In this work, we have used computational methods to predict the binding epitopes on DENV NS1 for three mAbs. We first showed DENV serotype specificity by ELISA. Secondly, we identified potential epitopes for the published mAbs 1H7.4 and 1G5.3 and compared these with published experimental results, providing validation of the prediction approach. Finally, we applied our approach to epitope prediction for a previously unpublished mAb, Gus2, which was shown experimentally to be cross-reactive against NS1 from all four DENV serotypes.

## 2. Results

### 2.1. Specificity of Mabs against DENV

**Figure 1:**
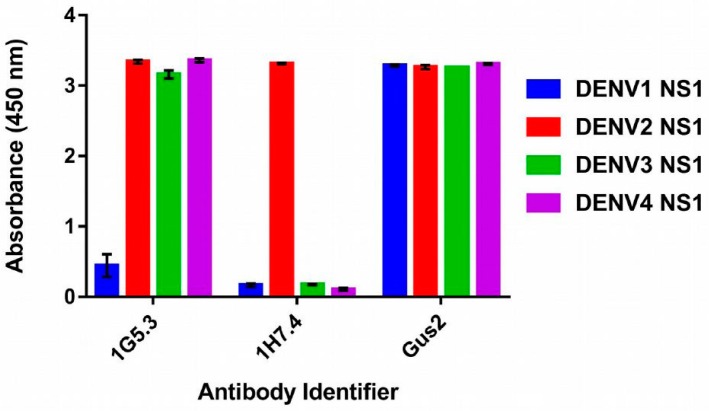
Antibody recognition of recombinant DENV NS1 by ELISA. 1G5.3, 1H7.4 and GUS2 antibodies were added to wells with immobilized recombinant DENV NS1. Binding was detected with a secondary HRP-conjugated antibody against mouse IgG.

Figure 1 shows the serotype specificity of monoclonal antibodies 1G5.3, 1H7.4 and GUS2 against immobilized recombinant DENV NS1 from each serotype. In an ELISA format, 1G5.3, 1H7.4 and GUS2 antibodies were incubated with immobilized recombinant NS1 from each DENV serotype and binding was detected with HRP-conjugated anti-mouse IgG antibody. The results show that 1H7.4 is serotype specific for DENV2 NS1 protein, in agreement with published results [19]. Antibody 1G5.3 was found to recognize NS1 proteins of three serotypes DENV2–4. This is somewhat in contrast to previous experimental data in which DENV2 and DENV4 bound strongly to the antibody, but DENV3 only bound weakly [19]. This could reflect either a different folding of the recombinant NS1 compared to the native NS1 used in that study, or a lower concentration of DENV3 NS1 isolated from the infected Vero cells. For the GUS2 antibody, pan-reactivity to NS1 from all four serotypes of DENV was observed. The observed specificity of these antibodies to the Dengue virus serotypes was explored against the epitope calculations given in the following sections.

### 2.2. Recognition of DENV1–4 NS1 by Antibody 1H7.4

The epitope identified experimentally for 1H7.4 is referred to as LD2 (residues 25–33) [19]. Firstly, we used our approach to identify epitopes of DENV recognized by 1H7.4. The antibody structure was built using the crystal structures of homologous antibodies from PDB entries “1SY6”, “1Z3G” and “3S62” as templates.

**Figure 2:**
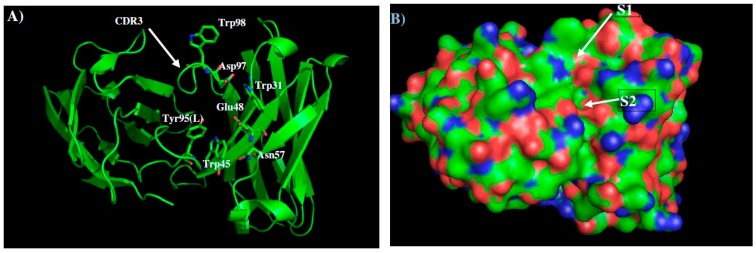
Model structure (**A**) and surface presentation (**B**) of 1H7.4 built by homology modeling. The contributing residues around CDR3 loop are highlighted in stick. The positively charged, negatively charged and hydrophobic surface regions are colored in blue, red and green in panel B. Two binding sites S1 and S2 are labeled. See context for the details.

Figure 2 shows the model structure and surface of the 1H7.4 with the contributing CDR residues highlighted (Figure 2A). Two distinct negatively charged regions, S1 and S2 are identified around the CDR3 loop. Region S1 is formed by H chain residues Trp31, Asp97, Trp98 and by the L chain residue Tyr95, and region S2 by H chain residues Trp45, Glu48, Asn57. These two binding surfaces are separated by ca. 5.0 Å. In addition, minima were also identified in a groove formed by the light chain residues Tyr48, Trp90, Tyr31, His33 and Arg30. However, the flexibility of Arg30 and Tyr31 side chains hinder the accessibility of this groove, so the MCSS minima in this region were discarded.

**Figure 3:**
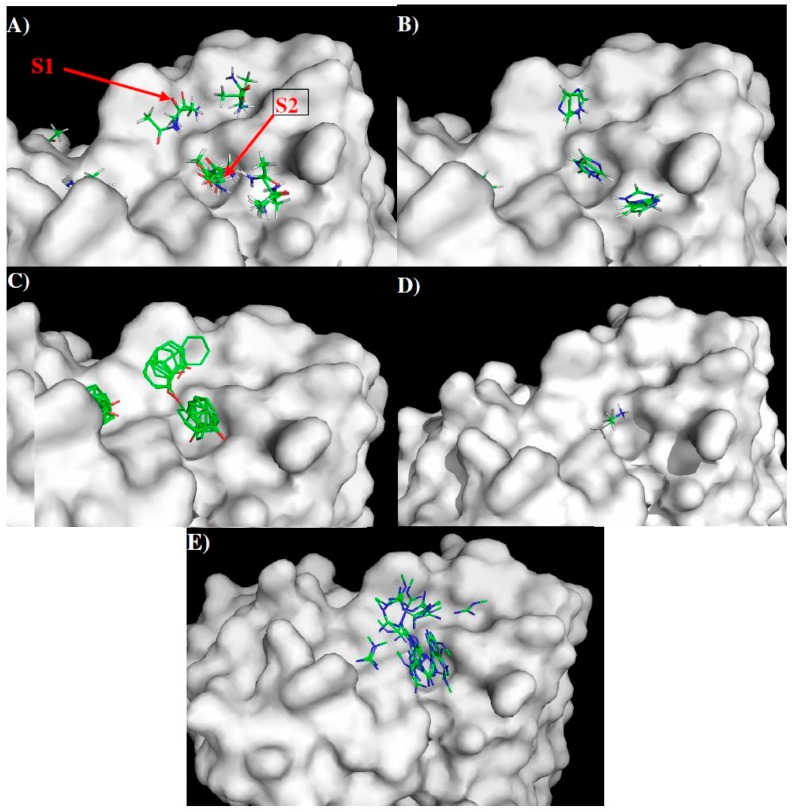
Selected MCSS minima of functional groups on the surface of 1H7.4. (**A**) ACEM; (**B**) IMIA; (**C**) PHEN; (**D**) MAMM; (**E**) MGUA. Two binding sites S1 and S2 are also labeled. See context for the details.

Figure 3 shows the distribution of MCSS minima of functional groups on the surface of 1H7.4. Overall, the distributions of the MCSS minima closely correspond to the physical properties of the surfaces with important specific interactions to residues within the heavy chain. For apolar groups such as MESH and IBUT (small group) and BENZ (aromatic rings), no minima were found due to the highly charged nature of the surface around the CDR3 loop. For the polar groups, ACEM minima were identified with two clusters in S1 forming hydrogen bonds to Asp97 and to the carbonyl oxygen of Ser29 of the H chain, respectively. On S2, 11 ACEM minima were found hydrogen bonding to Glu48 and the NE group of the H chain Arg55. For the IMIA group, a total of seven minima were found with direct interactions to heavy chain residues in S1 and S2. These consist of two minima interacting with Asp97 (H chain) and the carbonyl oxygen of Ser29 (H chain) in S1, two minima forming π–π interaction to Trp31 (H chain) in S1, and three minima interacting with Glu48 and NE of Arg55 (both of H chain) in S2. For the PHEN group, 12 minima were found grouped at S1 and S2 with one cluster forming a π–π interaction to Trp98 and hydrogen bonding to Asp97 (both of H chain) on S1 and the other to Trp31 and Glu48 of H chain on S2.

No ACET minima were found due to the negatively charged nature of the surfaces. While only one MAMM minima was found on S2 interacting with Asp97 (H chain), 35 MGUA minima were distributed over S1 and S2, interacting with negatively charged residues Asp97 and Glu48 of the H chain. The best minima of MGUA were located close to Glu48 (H chain) of S2.

Using the minima on the two surfaces S1 and S2, we constructed a sequence pattern for the peptides that could potentially bind to the antibody. The maximum distance between the two binding surfaces S1 and S2 is approximately 6.0 Å, indicating an adjacent position. While MGUA, IMIA, PHEN, and ACEM minima were found on both surfaces S1 and S2, only one MAMM minima was found on the surface S2. Therefore, the key sequence pattern for the binding epitope peptides can be defined as “XZ”, in which X = R, H, Y, Q, N, and Z = K, R, H, Y, Q, N. Table 1 lists the distribution of key MCSS minima and its derived sequence pattern for antibody 1H7.4.

The sequence pattern was subsequently used to search for “binders” (see Materials and Methods) from the peptide libraries derived from the sequence of the DENV2 NS1 protein. The seven libraries were searched for peptides matching the calculated sequence pattern of the binding epitope. There are 352 residues in DENV2 NS1, resulting in 50 × 7-mer peptides for each of seven libraries. Appendix S1 in the Supplementary Materials shows the identified binder peptides from each set of peptide libraries with the binders displayed in lower case characters. Overall, eleven peptides were identified with potential to bind to the antibody (named 1H7.4-Pn, *n* = 1 to 11 in Table 2). Analysis of the surface location of these binder peptides using the crystal structure of DENV2 NS1 protein showed that some of these peptides could be eliminated since, even though they are exposed, form either turn conformation (peptide 1H7.4-P1), or a long helical conformation (peptide 1H7.4-P3). Several other peptides buried within the protein or in the dimer interface were eliminated (for details see Figure S1 in Supplementary Materials). Table 2 summarizes the analysis results of these regions.

**Figure 4:**
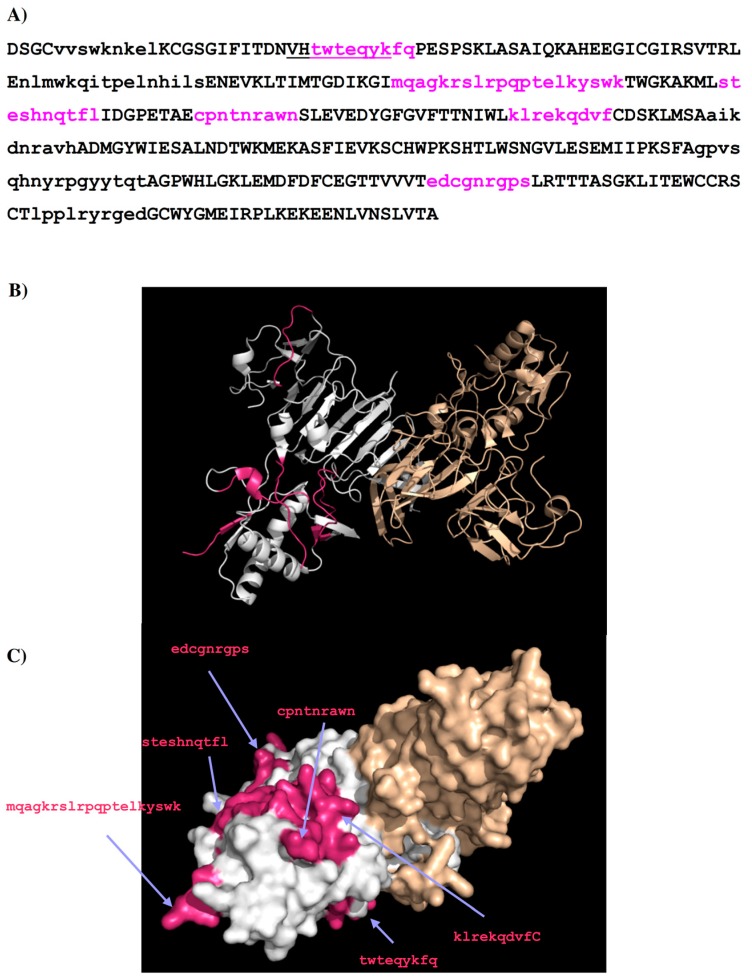
(**A**) The predicted epitopes of DENV2 NS1 protein to antibody 1H7.4 are highlighted in lower case and colored magenta in the protein sequence. The peptides identified as binders using the sequence search only are shown in lower case. The selection criteria based on their surface exposure and secondary structure is described in detail in the supplementary section (Figure S1). The epitope LD2 is highlighted with underline; (**B**) Backbone presentation of the dimer form of DENV2 NS1 protein showing the predicted epitopes in magenta; (**C**) Surface presentation of the dimer form of DENV2 NS1 protein showing the predicted epitopes in magenta.

As a result, only six binder peptides are considered as potential epitopes for the antibody, i.e., **1**—residues 27–35 “TWTEQYKFQ”; **2**—residues 97–116 “MQAGKRSLRPQPTELKYSWK”; **3**—residues 125–134 “STESHNQTFL”; **4**—residues 143–151 “CPNTNRAWN”; **5**—residues 170–178 “KLREKQDVF”; **6**—residues 289–297 “EDCGNRGPS”. Figure 4 shows the DENV2 NS1 sequence with these epitopes highlighted in lower case and colored in magenta (Figure 4A), their positions in the protein structure (Figure 4B) and on the protein surface (Figure 4C). Epitope **1** overlaps with the epitope LD2 (residues 25–33 “VHTWTEQYK”), experimentally identified previously [19].

**Figure 5:**
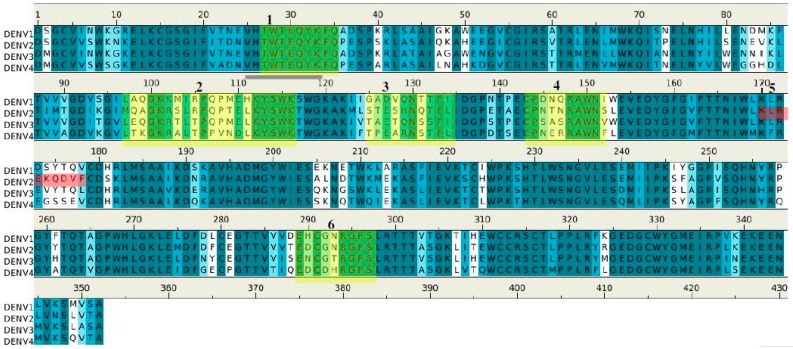
Sequence alignment of DENV serotypes (**1**–**4**) with the six (**1**–**6**) epitopes for the recognition of antibody 1H7.4. The boxes show the five epitopes that are conserved across the DENV1–4 (coloured in yellow/green), and the also the critical epitope responsible for the selectivity of 1H7.4 against the serotype DENV2 (colored in red). The epitope LD2 is highlighted with underline.

Figure 5 shows the location of the six potential epitopes of NS1 proteins in all four dengue virus serotypes predicted to bind to antibody 1H7.4. While five of the epitope peptides (i.e., **1**–**4** and **6**) were located at conserved sequence positions amongst all four serotypes (coloured in yellow/green), significantly, one epitope, epitope **5**, was found only in DENV2 (colored in red). Epitope **1** overlaps with peptide LD2 and is conserved amongst all four DENV serotypes. However experimental binding data ([19] and replicated here) shows that 1H7.4 is selective for DENV2 NS1. This suggests a role for additional conformational features of NS1 that influence epitope specificity with predicted epitope **5** (unique to DENV2) potentially playing a previously unrecognized role in the 1H7.4 selectivity, especially as epitope **1** is considered to be close to the membrane in the dimeric complex of DENV2 NS1 [20].

### 2.3. Recognition of DENV NS1 to Antibody 1G5.3

**Figure 6:**
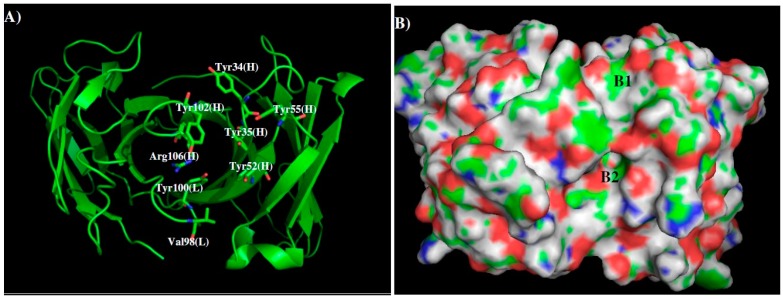
The model structure (**A**) and surface presentation (**B**) of antibody 1G5.3. The contributing residues around CDR3 loop are highlighted in stick. The positively charged, negatively charged and hydrophobic surface regions are colored in blue, red and green in panel B. Two binding sites B1 and B2 are labeled. See context for the details.

We created a 3D model of the VL and VH domains of antibody 1G5.3 using the crystal structures of the antibodies from PDB entries “1KEN”, “3J1S”, “4HC1”, “4BKL”, “2AAB” and “1MF2” as templates. Figure 6 shows the model structure and surface with the contributing CDR residues highlighted (Figure 6A).

Compared to the 1H7.4 antibody, the shape of the surface around the CDR3 loop is more defined with a hydrophobic surface B1 formed by four tyrosine residues (Tyr34, Tyr35, Tyr102, Tyr55) of the H chain, and a pocket B2 formed by L chain (Val98, Tyr100) and H chain (Tyr35, Tyr52, Arg106) residues. The positively charged Arg106 of the CDR3 loop sits at the gate of the pocket, resulting in the charged nature of B2. Similar to 1H7.4, a groove is formed between the L and H chains by L chain (Thr34, Val36) and H chain (Tyr102, Thr104) residues. Compared to the pocket B2, this groove is comparatively narrow for antigen access. Therefore, we discarded the MCSS minima inside this groove.

**Figure 7:**
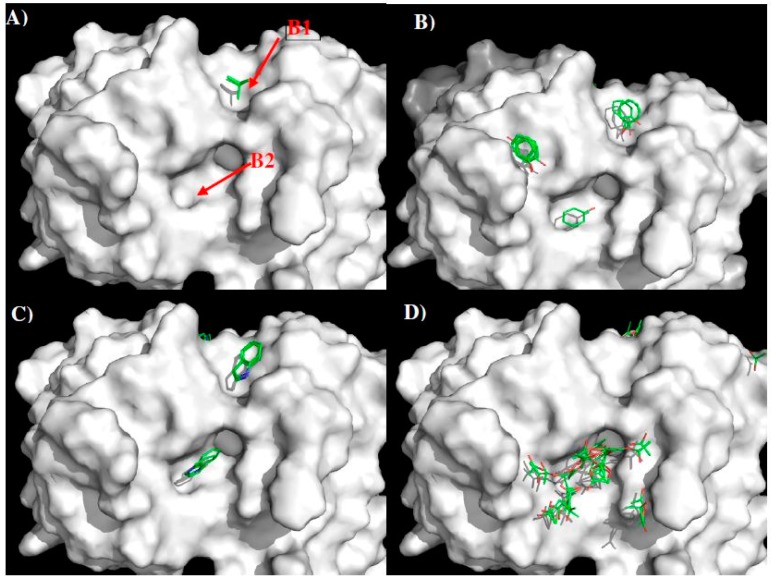
Selected MCSS minima of functional groups on the surface of 1G5.3. (**A**) IBUT; (**B**) PHEN; (**C**) INDO; (**D**) ACET. Two binding sites B1 and B2 are also labeled.

Figure 7 shows the locations of the minima of functional groups on the surface of 1G5.3. Overall, the minima are clustered around the binding sites B1 and B2. No minima of ACEM, MEOH, MESH, BENZ and IMIA were found on the surfaces around the CDR3 loop. For the apolar groups, three IBUT minima were identified at the center of the surface B1 by hydrophobic packing to the aromatic rings of four tyrosines. The interaction energies are calculated to be −11.00 kcal/mol. For the aromatic groups, five PHEN minima were located in B1 by forming hydrogen bonding to Tyr35 and Tyr55 of H chain with energies of (−12.00 to −12.30) kcal/mol, while one minimum is located in B2 by both hydrophobic interaction to Val98 and hydrogen bonding to Tyr52 (H chain) with the total interaction energy of −12.50 kcal/mol. For the INDO group, three minima were located on B1 by hydrogen bonding to Tyr35 of the H chain, and two minima in the pocket B2 by interacting with Tyr100 of the L chain. The corresponding interaction energies were calculated to be (−15, −15, −15.1) kcal/mol and (−15.1, −15.3) kcal/mol, respectively. While no MAMM and MGUA minima were found around the CDR3 loop because of the positively charged residue Arg106 (H chain), 43 ACET minima were distributed over the pocket B2 largely due to their strong electrostatic interactions with Arg98 of the L chain. The best minimum in fact was found to specifically interact with Tyr100 of the L chain with an interaction energy of −19 kcal/mol.

Based on the distribution of the important minima shown in Figure 7, a sequence pattern for peptides that bind 1G5.3 was derived. The MCSS minima at binding sites B1 and B2 are separated by ca. 11.5 Å, a distance that could accommodate a gap of two amino acids. While only ACET minima were obtained at B2 exhibiting interactions with Arg106 of the H chain, only small apolar group IBUT were located on B1. Aromatic groups PHEN and INDO were on both B1 and B2. Therefore, the key sequence pattern for the binders was defined as “X–Z”, in which X = (I or L or V), Y, W, and Z = Y, W, (D or E). Table 3 lists the distribution of key MCSS minima and the derived sequence pattern for antibody 1G5.3.

**Figure 8:**
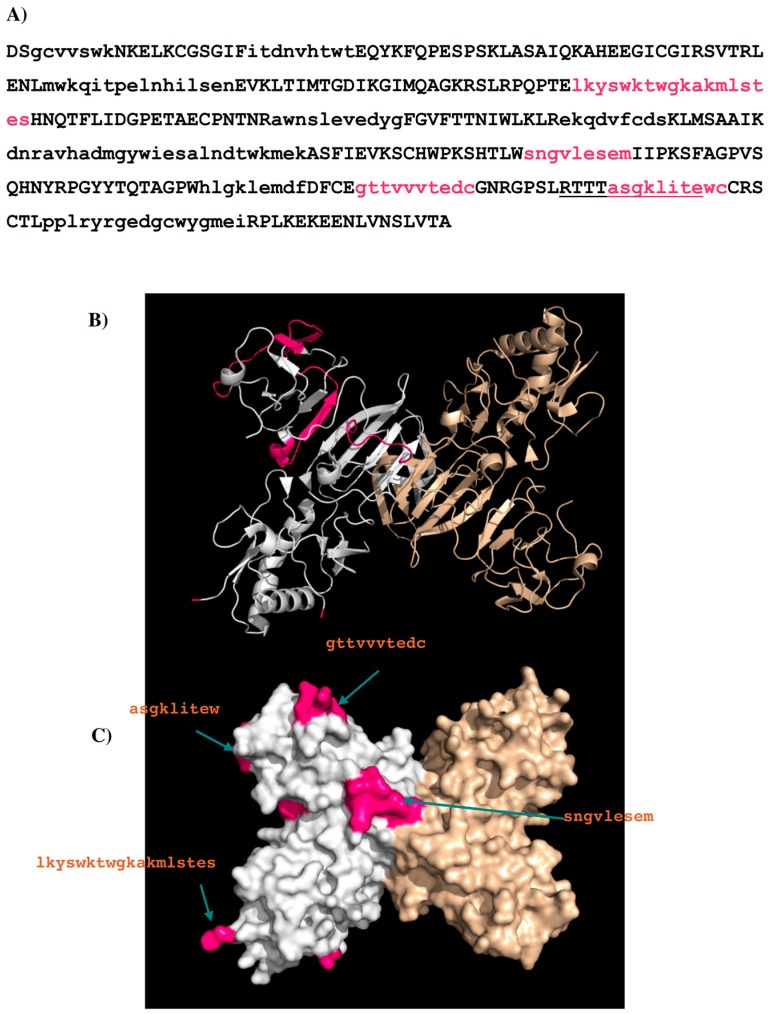
(**A**) The predicted epitopes of DENV2 NS1 protein to antibody 1G5.3 are highlighted in lower case and colored magenta in the protein sequence. The peptides identified as binders using the sequence search only are shown in lower case. The selection criteria based on their surface exposure and secondary structure is described in detail in the supplementary section (Figure S2). The epitope 24C is highlighted with underline; (**B**) Backbone presentation of the dimer form of DENV2 NS1 protein showing the predicted epitopes in magenta; (**C**) Surface presentation of the dimer form of DENV2 NS1 protein showing the predicted epitopes in magenta.

Seven sets of peptide libraries were generated. Appendix S2 in the Supplementary Materials shows the identified binders from each of the seven sets of peptide libraries with the binder residues highlighted in lower case. In total, 12 peptides were identified with potential binding to the antibody 1G5.3 (named 1G5.3-P*n*, *n* = 1 to 12 in Table 4). Based on the surface analysis (for details see Figure S2 in Supplementary data), only four peptides are selected as potential epitopes for the antibody 1G5.3. These are **I**—residues 111–128 “LKYSWKTWGKAKMLSTES”; **II**—residues 233–241 “SNGVLESEM”; **III**—residues 282–291 “GTTVVVTEDC”; and **IV**—residues 303–311 “ASGKLITEWC”. Figure 8 shows the DENV2 NS1 sequence with these epitopes highlighted in lower case and colored in magenta (Figure 8A), their positions in the protein structure (Figure 8B) and on the protein surface (Figure 8C). Epitope **IV** overlaps with the epitope 24C (residues 299–309 “RTTTASGKLIT”) observed experimentally [19].

**Figure 9:**
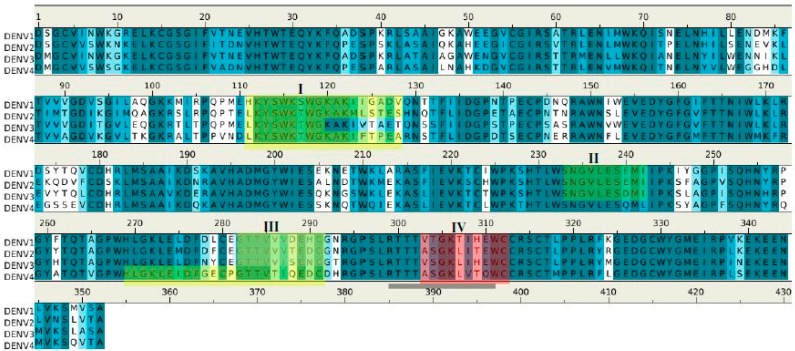
Sequence alignment of DENV serotypes (**1**–**4**) with the 4 (**I**–**IV**) epitopes for the recognition of antibody 1G5.3. The boxes (colored in green/yellow) highlight the epitopes of each serotype and the critical epitopes responsible for the selectivity of 1G5.3 against the serotypes DENV2/DENV4 are colored in red. The boxes show the five epitopes that are conserved across the DENV1–4 (coloured in yellow), and the also the critical epitope responsible for the selectivity of 1G5.3 against the serotype DENV2 (colored in red). The epitope 24C is highlighted with underline.

Figure 9 shows the epitopes of NS1 proteins of four dengue virus serotypes (DENV1–4) predicted to bind to antibody 1G5.3.

Epitopes **I** and **II** are located at largely conserved positions amongst the sequences of the serotypes, however, epitopes **III** and **IV** show (significantly) more variation. These two epitopes are in close proximity, located towards the C terminus, thus forming a potential binding site (Figure 8C). Interestingly, epitope **II** is missing in DENV4; however, this could be compensated by the longer epitope **III** (23 mer) found in DENV4. The selectivity of 1G5.3 to DENV2–4 could thus be mainly due to the sequence variability at epitope **IV** which is overlapping with the epitope 24C (13). In particular, residue Thr308 of epitope **IV** in DENV1 is replaced by the hydrophobic Leu in DENV2–4. This residue is completely exposed to solvent and could thus serve as the key residue for antibody-antigen recognition.

### 2.4. Prediction of Epitopes of DENVs to Antibody GUS2

**Figure 10:**
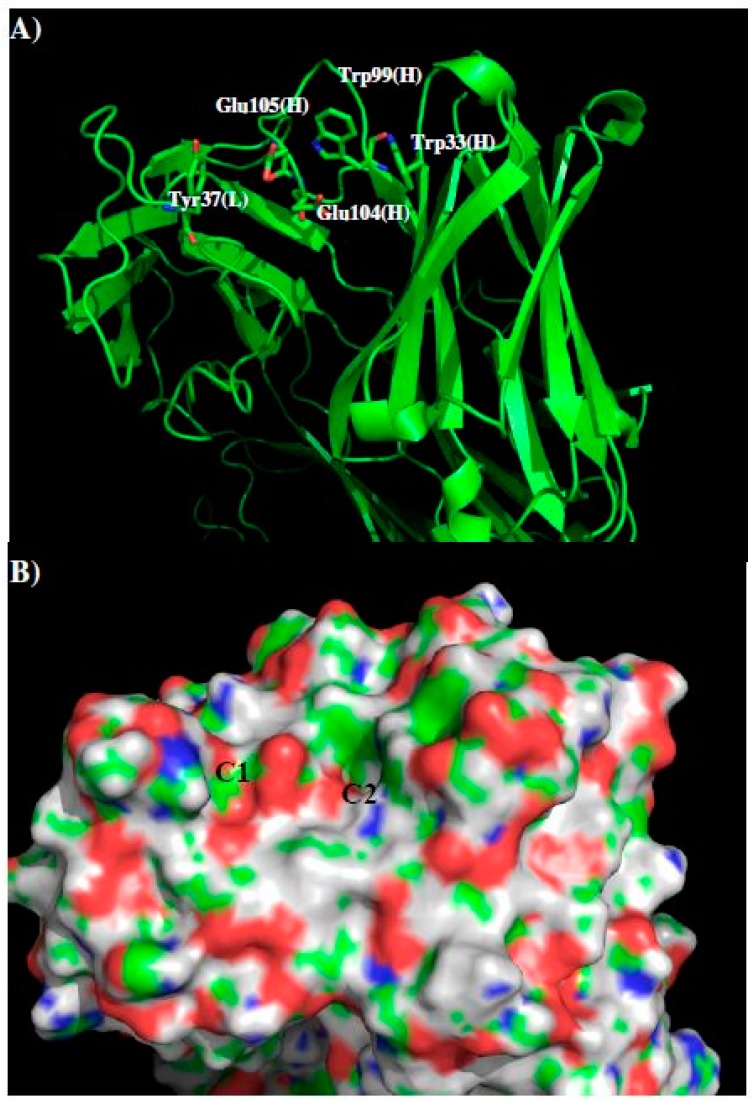
The model structure (**A**) and surface presentation (**B**) of antibody GUS2. The contributing residues around CDR3 loop are highlighted in stick. The positively charged, negatively charged and hydrophobic surface regions are colored in blue, red and green in panel B. Two binding sites C1 and C2 are labeled. See context for the details.

GUS2 is a previously unpublished antibody which binds to all four of the dengue virus serotypes, as demonstrated by ELISA against recombinant NS1 from each serotype (Figure 1). Here, we used our approach to predict the epitopes of DENV1–4 that are identified by GUS2. We built the model structure of GUS2 using crystal structures of the antibodies from PDB entries “1UZ6”, “1UZ8”, “3CFB”, “1FOR”, “1RVF” and “3DIF” as templates. Figure 10 shows the model structure and surfaces of GUS2 with the contributing CDR residues highlighted (Figure 10A). Overall, the landscape around the CDR3 loop is relatively flat with two negatively charged residues Glu105 and Glu106 of H chain located at the center of the surface. Residues Tyr37 of L chain and Glu105 of heavy chain form a binding site C1. In addition, a small cavity C2 is identified ca. 5.50 Å away from C1, formed by Trp99 and Trp33 of H chain.

**Figure 11:**
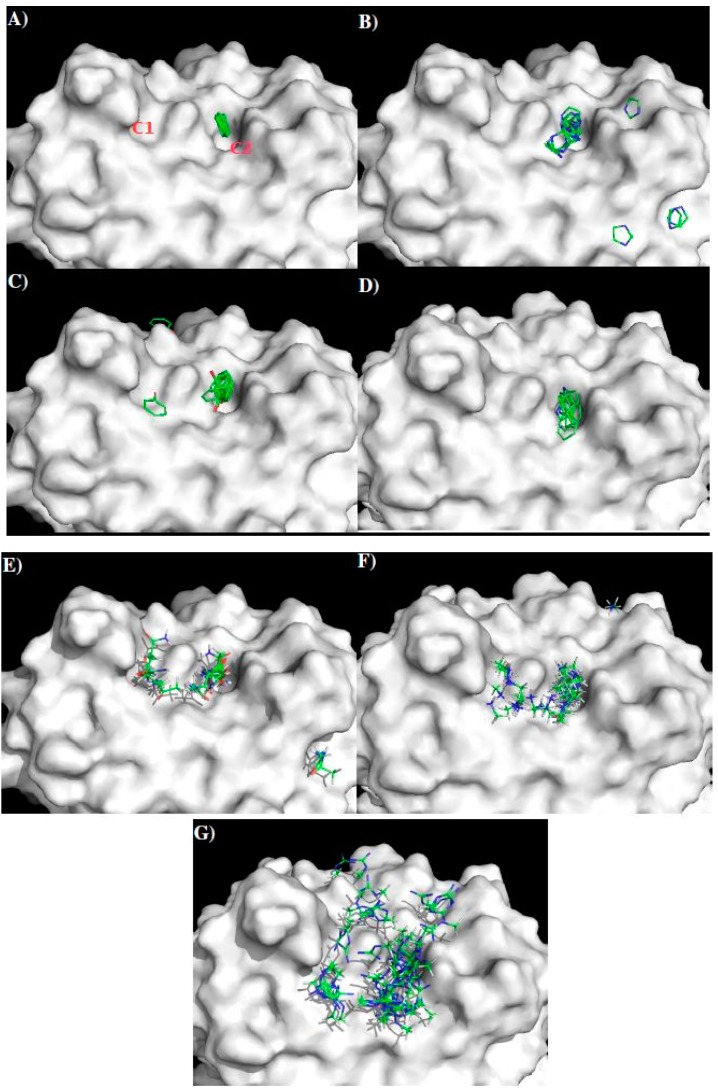
Selected MCSS minima of functional groups on the surface of GUS2. (**A**) BENZ; (**B**) IMIA; (**C**) PHEN; (**D**) INDO; (**E**) ACEM; (**F**) MAMM; (**G**) MGUA. Two binding sites C1 and C2 are also labeled. See context for the details.

Figure 11 shows the distribution of MCSS minima of functional groups on the surface of GUS2. For the small groups, no MEOH, MESH and IBUT minima were found due to the highly charged nature of the surface around the CDR3 loop. For the aromatic rings such as BENZ, IMIA, PHEN and INDO, the minima were concentrated at the cavity C2, by hydrophobic interaction to residues Trp99 and Trp33 of the H chain. Overall, 14 BENZ minima, 41 IMIA minima, 15 PHEN minima and 5 INDO minima were found in the cavity C2, while only one PHEN minima was detected on the surface C1. The best minima have interaction energies of −10.9 kcal/mol for BENZ, −11.6 kcal/mol for IMIA, −14.1 kcal/mol for PHEN and −12.7 kcal/mol for INDO. For the polar group ACEM, 45 minima span C1 and C2 with two clusters interacting with Glu105 and Glu106 of the H chain, respectively. A similar pattern was obtained for the positively charged groups MAMM and MGUA, with 70 MAMM and 50 MUGA minima around CDR3. The best minima of MAMM and MGUA were at the site C1 with interaction energies of −20.1 kcal/mol and −20.5 kcal/mol. No ACET minima were found due to the negatively charged nature of the surfaces.

Using the minima on the two sites C1 and C2, we constructed a sequence pattern for the peptides that could potentially bind to the antibody. The maximum distance between the two binding sites C1 and C2 is approximately 5.5 Å, indicating that there is no amino acid gap between the two sites. While MGUA, IMIA, PHEN, and ACEM minima were found on the surfaces C1 and C2, only one MAMM minima is found at C2. Therefore, the key sequence pattern for the binding epitope peptides can be defined as “XZ”, in which X = R, K, Q, N, Y, and Z = R, K, Q, N, Y, F, H or W. Table 5 lists the distribution of key MCSS minima and its derived sequence pattern for antibody GUS2.

Appendix S3 in the Supplementary Materials shows the identified epitopes from each set of peptide libraries with the epitopes highlighted in lower case characters. In total, 10 peptides were identified as potential binders to the antibody GUS2 (named GUS2-Pn, *n* = 1 to 10 in Table 6). 

**Figure 12:**
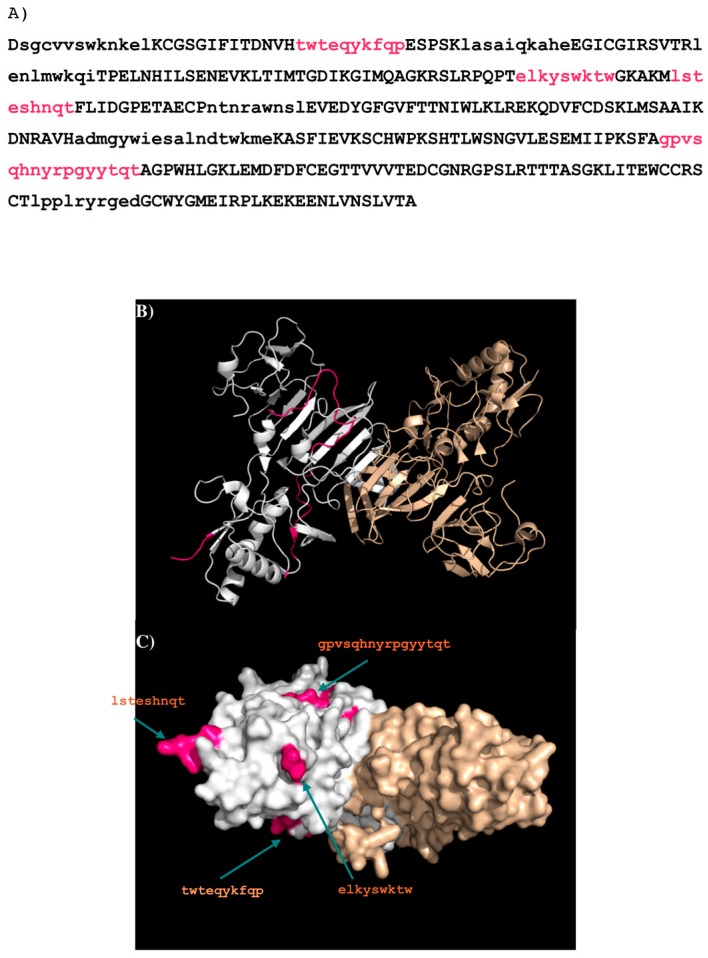
(**A**) The predicted epitopes of DENV2 NS1 protein to antibody GUS2 are highlighted in lower case and coloured magenta in the protein. The peptides identified as binders using the sequence search only are shown in lower case. The selection criteria based on their surface exposure and secondary structure is described in detail in the supplementary section (Figure S3); (**B**) Backbone presentation of the dimer form of DENV2 NS1 protein showing the predicted epitopes in magenta; (**C**) Surface presentation of the dimer form of DENV2 NS1 protein showing the predicted epitopes in magenta. Note that the majority of the loop (residues 108–128) is missing in the crystal structure of DENV2 NS1 protein (PDB code 4O6B) so that the epitope B “elkyswktw” and epitope C “lsteshnqt” are only illustratively displayed.

Further analysis of the surface accessibility and conformations of these peptides (for details see Figure S3 in supplementary) resulted in the selection of only four epitopes to bind the antibody (i.e., **A**—residues 27–36 “TWTEQYKFQP”; **B**—residues 110–118 “ELKYSWKTW”; **C**—residues 124–132 “LSTESHNQT”; **D**—residues 249–264 “GPVSQHNYRPGYYTQT”). Figure 12 shows the DENV2 NS1 sequence with these epitopes highlighted in lower case and colored in magenta (Figure 12A), their positions in the protein structure (Figure 12B) and on the protein surface (Figure 12C). Epitope A and epitope B overlap with LD2 and LX1 respectively.

**Figure 13:**
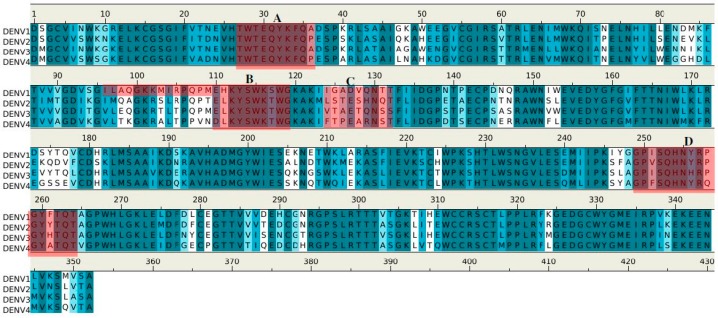
Sequence alignment of DENV serotypes (**1**–**4**) with the four (**A**–**D**) epitopes for the recognition of antibody GUS2. The boxes highlight the epitopes of each serotype. All the epitopes are coloured in red as GUS2 is a non-selective antibody against the dengue virus serotypes.

Figure 13 shows the epitopes of NS1 proteins of the four dengue virus serotypes (DENV1–4) predicted to bind to antibody GUS2. The epitopes are found at conserved positions amongst all serotypes with the exception of epitope **C**. It is surprising to identify a non-conserved epitope, given the non-selective nature of antibody GUS2. The lack of effect on selectivity of epitope **C** can probably be explained by examining the location of the epitope. Epitope **C** is in close proximity to epitope **B**, and together they combine to form a long, protruding loop (Figure 12B). The binding propensity of this region is therefore limited because of the length and resulting flexibility of the loop. Note that the majority of the loop (residues 108–128) is missing in the crystal structure of DENV2 NS1 protein (PDB code 4O6B [20] so that the epitope **B** and **C** are only illustratively displayed in Figure 12B,C.

The epitope LX1 (overlapping with epitope **B**) has previously been shown to bind to mAbs which are cross-reactive against all four serotypes of DENV [19]. In this study, the LX1 peptide was tested for binding to Gus2 by ELISA, and verified that it does indeed bind to Gus2 (data not shown). This confirms the involvement of epitope B predicted in this study.

## 3. Materials and Methods

Our paper presents a method for the computational prediction of antibody-binding epitopes on antigens. The minimum information our method requires is the amino acid sequences of the antibody and the antigen. The accuracy of our prediction is increased, if the 3D structures of the antigen and/or antibody are known as well. The method begins with the creation of an homology model of the antibody in question based on its sequence. While all antibodies show comparatively high sequence identity which facilitates the modeling process significantly, care needs to be taken when modeling the CDR regions. We have shown, however, that our results do not depend significantly on the exact conformation of the CDR loops [11]. In a second step, extensive MCSS calculations (see details below, and [16]) are performed using small chemical fragments corresponding to functional groups of amino acids. The MCSS method probes the antigen binding pocket of the antibody for favorite locations (“minima”) of amino acid side chains. In the third step, we select suitable minima of chemical fragments on the binding sites of the antibody. The pattern of the selected MCSS minima distribution is then converted into a sequence pattern characteristic for the binding of the antigen to the antibody. In the fourth step, this sequence pattern is then used as a “fingerprint” to search through the sequence of the antigen for those peptide regions with a significant occurrence (see [11]) of the fingerprint. The peptide regions are then considered as possible epitopes that can bind to the antibody. The details of our method are described in [11], however, we summarize the single steps in the paragraphs below, as well as the experimental methods we used.

### 3.1. Antibodies

The mAbs 1H7.4, 1G5.3 and Gus2 are derived from mouse hybridomas as previously described [21]. Briefly, BALB/c mice were immunized with 10 µg of purified soluble DENV2 NS1 protein. After a final boost, splenocytes were isolated and fused with SP2/O cells. Positive clones were selected by immobilized NS1 Enzyme Linked Immunosorbent Assay (ELISA). The sequences of the variable heavy and light chains were obtained by RT-PCR amplification from hybridoma mRNA. Briefly, total mRNA was extracted from hybridoma cell pellets using RNeasy Mini Kit (Qiagen, Melbourne, Australia). RT-PCR was performed using SuperScript III First-strand synthesis kit (Life Technologies, Mulgrave, Australia) with Oligo(dT) primers. Variable heavy and light chain amplification was achieved using the primer set described in Brocks et al. [22] and amplicons of the correct size were sub-cloned into pGemT-easy vector prior to Sanger sequencing.

### 3.2. Enzyme Linked Immunosorbent Assay

Binding of antibodies to recombinant DENV NS1 was confirmed by direct binding ELISA. Purified recombinant NS1 from each of the four serotypes (200 µL, 3 µg/mL) was coated per well on a Nunc MaxiSorp plate (Life Technologies, Mulgrave, Australia), in PBS at 4 °C overnight. The plate was washed three times with phosphate buffered saline (PBS) with 0.01% Tween20 (PBS-T). The plate was blocked using PBS with 2% skim milk powder (MPBS). After blocking, 200 µL of 3 µg/mL test antibody diluted in MPBS was added to relevant wells. Following one hour incubation, plates were washed three times with PBS-T and probed for one hour with 200 µL of HRP-conjugated goat anti mouse IgG H+L (Life Technologies) in MPBS at 0.1 µg/mL. Three final washes were performed with PBS-T. 100 µL TMB substrate (Sigma Aldrich, Sydney, Australia) was added to develop the chromogenic signal. The reaction was stopped with 2N sulphuric acid and the absorbance at 450 nm was read using the Spectramax (Molecular Devices, Sunnyvale, CA, USA).

### 3.3. Homology Modeling of the Antibodies

The sequences of the antibodies 1H7.4, 1G5.3, and GUS2 were used to search for the closest related antibody with known 3D structure using a BLAST search against sequences of proteins deposited in the protein data bank (http://blast.ncbi.nlm.nih.gov). For the 1H7.4 mAb, the most homologous sequences were from PDB entries “1SY6” (Crystal Structure of CD3γε Hetero dimer in Complex with OKT3 Fab Fragment [23]), “1Z3G” (Crystal structure of complex between Pvs25 and Fab fragment of malaria transmission blocking antibody 2A8 [24]) and “3S62” (Structure of Fab fragment of malaria transmission blocking antibody 2A8 against P. vivax P25 protein [25]). The VL and the VH domains of 1H7.4 show sequence identities of (95.30%, 91.20%) to 1SY6, (96.20%, 84.00%) to 1Z3G, and (98.10%, 89.6%) to 3S62, respectively. For the 1G5.3, mAb the best matching antibody sequences found were from entries “1KEN” (Influenza virus hemagglutinin complexed with an antibody [26]), “3J1S” (Structure of adeno-associated virus-2 in complex with neutralizing monoclonal antibody A20 [27]), “4HC1” (Crystal structure of a loop deleted mutant of human MAdCAM-1 D1D2 complexed with Fab 10G3 [28]), “4BKL” (Crystal structure of the arthritogenic antibody M2139 (Fab fragment) in complex with the triple-helical J1 peptide [29]), “2AAB” (Structural basis of antigen mimicry in a clinically relevant melanoma antigen system [30]), and “1MF2” (Anti HIV1 protease FAB complex [31]). The amino acid identities of the VL and VH domains of the 1G5.3 are (75.2%, 87.7%) to 1KEN, (76.1%, 83.6%) to 3J1S, (75.2%, 86.9%) to 4HC1, (98.2%, 65.0%) to 4BKL, (96.5%, 63.5%) to 2AAB and (93.8%, 60.9%) to 1MF2, respectively. As for the GUS2 mAb, the PDB entries of the templates were “1UZ6/1UZ8” (Anti-Lewis X FAB fragment [32]), “3CFB” (High-resolution structure of blue fluorescent antibody EP2–19G2 in complex with stilbene hapten at 100K [33]), “1FOR” (Structure determination of an Fab fragment that neutralizes human rhinovirus 14 [34]), “1RVF” (Fab complexed with intact human rhinovirus [35]), and “3DIF” (Crystal structure of FabOX117 [36]). The amino acid identities of the VL and VH domains of the GUS2 are (98.2%, 67.5%) to 1UZ6/1UZ8, (97.3%, 63.3%) to 3CFB, (66.4%, 88.3%) to 1FOR, (66.4%, 88.3%) to 1RVF and (71.7%, 85.0%) to 3DIF, respectively. All the model constructions were carried out with the Modeller software including explicit optimization of the Complementary Determining Regions (CDR) loop regions as implemented in Modeller [37].

### 3.4. MCSS of Functional Groups

Using the homology model of an antibody, our quCBit software (http://www.computistresearch.com) implementing our MCSS approach was used to scan the preferred locations of functional chemical groups on the binding surfaces around the “Complementary Determining Regions” (CDRs). Eleven functional groups were used, each of which corresponds to the side chains of different amino acids [16,38] (Table 7). All the parameters for both protein and functional groups were taken from the CHARMM22 all-hydrogen atom force field [39].

Three hundred replicas of each functional group were randomly distributed inside a 12-Å radius sphere around the CDRs. The details of the CDR loop conformations have been shown to be insignificant for the distribution of MCSS minima and, we use only single conformation of the CDRs [10,11]. The CDRs are defined by (Tyr31, Tyr48, Ser55, Ser91, Asn93) of L chain and (Trp31, Trp45, Asn56, Lys72, Arg96, Trp98, Ser99, Tyr103) of H chain for the 1H7.4 mAb; (Leu36, Tyr53, Ser60, Ser95) of L chain and (Ser32, Tyr34, Tyr35, Tyr52, Tyr55, Thr75, Ser99, Tyr101, Tyr103, Tyr110) of H chain for 1G5.3 mAb; and (Ile35, Tyr54, Ser61, Leu97, Leu99) of L chain and (Trp33, Val37, Gln50, Ser58, Arg98, Gly100, Glu105, Phe108) of H chain for the GUS2 mAb. A 500-step MCSS was performed. During all the MCSS calculations, each replica only interacts with a target protein, and not with the other replicas. The details of MCSS calculations have been described previously [16,38] During the calculations, the non-bonded interaction was truncated at 20 Å and the dielectric constant was set to 10 to mimic solvent screening effects [40].

### 3.5. Identification of Sequence Pattern

Interaction energy of −10.00 or −12.00 kcal/mol was used as the threshold for the minima of polar and apolar functional groups, respectively, for the antibodies 1H7.4, 1G5.3 and GUS2. For the charged groups ACET, MAMM and MGUA, −15.00 kcal/mol were used. The spatial patterns of the locations of the MCSS minima on the surface of the antibody were converted into a sequence pattern according to the relationship between the functional groups and amino acids as given in Table 7. This sequence pattern will serve as the fingerprint to identify the epitopes of antigens.

### 3.6. Search for Epitopes Based on the Sequence Pattern

The sequence pattern obtained using the method described in Section 3.5 was used to identify candidate peptides from the sequence of dengue virus serotypes (DENV1–4). We divided the whole NS1 protein sequence into overlapping peptides of 7 amino acids in length (7-mer), allowing an efficient scan of the MCSS minima distributions of the average sized binding epitopes. To avoid artifacts by starting from a particular residue, the protein was sliced into 7-mer peptide libraries several times, starting from residue 1 up to 7. This results in seven libraries of 7-mer peptides. Each of the seven libraries was checked for sequence matches with the key pattern. The peptides with a sequence that matches the key pattern derived from MCSS minima of functional groups were considered to be potentially part of the epitope and labeled as the “binders”. Residues occurring in binder peptides from more than three libraries were considered part of the epitope. Therefore, the epitopes predicted from the seven sets of peptide libraries could vary in their length. The details of the epitope searching were described previously [10,11]. By searching the sequence of antigen only, some epitopes could be either in a helical conformation, or alternatively, buried inside the protein, so that these epitopes would be unlikely to bind antibodies. As the crystal structure of NS1 protein of DENV2 is available and DENV serotypes have very high sequence homology [20], we analyzed the surface accessibility of the epitopes in the NS1 dimer and selected those with significant surface accessibility as the final epitopes. The binder peptides of helical conformation, or embedded in the protein, or close to the other monomer were discarded.

## 4. Discussion

Previously, we described a new method to predict the binding epitopes of proteins to specific antibodies [10,11]. It involves three steps: (1) mapping of functional groups onto the surface of the antibody; (2) deriving a sequence pattern for potential binding peptides based on the distribution of significant minima of functional groups; and (3) searching the antigen sequence for binding peptides which are consistent with the sequence pattern. Here, we have applied this approach to identify binding epitopes of NS1 proteins of DENV serotypes to three independent antibodies, 1H7.4 and 1G5.3 [19] and GUS2. The recently solved crystal structure of the DENV2 NS1 protein [20] was used to further select the epitopes of significant surface accessibility for antibody binding. Overall, our results reproduced the epitopes identified for the previously characterized antibodies 1H7.4 and 1G5.3, and predicted a set of peptides that form epitopes for the non-characterized antibody GUS2. As a result, the amount of experimental work needed to identify antibody-binding epitopes for GUS2 is significantly reduced.

As well as the reproduction of known epitopes LD2 and 24C for the antibodies 1H7.4 and 1G5.3 [19], respectively, our calculations also present five additional epitopes at the regions of residues 97–115, 125–133, 143–151, 170–178, and 289–297 for the 1H7.4 (Figure 4) and three epitopes at the regions of residues 111–128, 233–241 and 282–291 for the 1G5.3 (Figure 8). While the additional epitopes for 1G5.3 are in the same locality as 24C thus forming potential conformational epitopes to the antibody (Figure 8B), some of the additional epitopes for 1H7.4 are found in different locations on the surface of the NS1 protein to the previously identified linear epitope, LD2 (Figure 4B). The crystal structure of dimeric NS1 [20] reveals that this linear epitope is close to a hydrophobic domain that is thought to interact with the cell membrane. However the recognition by this antibody of cell surface bound NS1 in immunofluorescence studies (data not shown) is not consistent with the steric hindrance expected of this location. Furthermore, the predicted epitope **1** (LD2; residues 25–33, sequence “TWTEQYKFQ”) is completely conserved across all four serotypes, despite the fact that 1H7.4 binds only to NS1 from DENV2. This implies that specific binding may be due to epitope recognition that is impacted by protein folding and conformational effects from other regions of the protein [19]. This is in agreement with our epitope mapping on different serotypes (DENV1–4). As shown in Figure 5, epitope **5** (residue 170–178, sequence “KLREKEQDVF”) is unique for DENV2 for the antibody 1H7.4, inferring the importance of this peptide on selectivity. The additional potential epitopes identified in this study for 1H7.4 antibody-antigen recognition will require further experimental validation.

For the antibody 1G5.3 (Figure 8), the predicted epitope **IV** or previously identified 24C (residues 299–309 “RTTTASGKLIT”) [19] is located at the C-terminus in the dimer form with full exposure to solvent and consequently to antibody; the other predicted epitopes consist of LD2 and LX1. This is in agreement with the previous experimental results in which the 1G5.3 reacts with non-reduced DENV2 NS1 in either the dimeric or monomeric forms [19]. As shown in Figure 9, the epitopes for the different serotypes were identified as conserved at the same position, except the sequences of epitopes **III** and **IV,** which are noticeably divergent amongst the serotypes. Moreover, these two epitopes are next to each other in the three-dimensional structure, forming an accessible recognition site (Figure 8C). Previously, Falconar et al. demonstrated that 1G5.3 is selective for DENV2/4, with weak binding to DENV3 [19], while our ELISA assay shows that it binds three serotypes DENV2–4. Considering that they identified epitope **IV** as the dominant site for recognition [19], the replacement of Thr308 (found in epitope **IV**) by Leu in only DENV1 is consistent with our experimental data that 1G5.3 is selective for DENV2–4.

Having verified the known epitopes and identified additional epitopes for the NS1 specific antibodies 1H7.4 and 1G5.3, and investigated the role that these individual epitopes play in binding, the method was then used to predict epitopes of antibody GUS2. The surface of the antibody around the CDR3 loop has a relatively flat topography with MCSS minima of different functional groups to those seen previously, with stronger interactions to the antibody (Figure 11). Four peptides (**A**–**D**) were predicted as the binding epitopes of GUS2, located at the regions of residues 27–36, 110–118, 124–132, and 239–264, respectively (Figure 12). In the four serotypes, epitopes **A** and **D** have significant sequence homology, whilst epitope **C** is divergent amongst the serotypes (Figure 13). Interestingly, epitopes **B** and **C,** which are adjacent in the linear amino acid sequence, combine together to form a long loop protruding from the crystal structure (Figure 12B). The conformational flexibility of the loop increases the entropy contribution thus reducing the binding affinity differences between the antibody and different antigens. This explains why the GUS2 antibody is non-selective as also demonstrated using ELISA, which shows that Gus2 binds the NS1 of all four DENV serotypes. Additionally, epitope **B** overlaps with epitope LX1, which is known to bind other mAbs against four serotypes of DENV NS1, and subsequent testing of LX1 showed positive binding to Gus2 (data not shown).

Overall, our calculations have identified several epitopes of the DENV NS1 protein to the antibodies 1H7.4, 1G5.3 and GUS2, and these peptides are in agreement with previously published (1H7.4 and 1G5.3) or experimentally validated (Gus2) results.

## 5. Conclusions

Previously we have developed a simple qualitative method to search for epitopes of proteins that bind to specific antibodies. In this work, we have applied this method to identify the recognition sites of antibodies 1H7.4, 1G5.3 and GUS2 to the NS1 proteins of the four dengue virus serotypes (1–4). While our calculations have reproduced the epitopes LD2 and 24C identified previously for the antibodies 1H7.4 and 1G5.3, respectively, additional potential epitopes were also obtained comprising residues 97–115, 125–133, 143–151, 170–178, and 289–297 for 1H7.4 and at residues 11–128, 233–241 and 282–291 for 1G5.3. Rather than the previously identified LD2, the selectivity of antibody 1H7.4 to DENV2 is most likely controlled by the residues 170–178 which is only found in DENV2. The binding of 1G5.3 to DENV2–4 can be explained by the sequence differences between DENV1 and DENV2–4 of the recognition region formed by the epitope at residues 282–291 and especially residues 303–311. For antibody GUS2, four epitopes are predicted at residues 27–36, 110–118, 124–132 and 249–264. The non-selectivity of GUS2 against the dengue virus serotypes is the result of the highly conserved sequence homology of all the NS1 epitopes in DENV1–4 (with the exception of epitope 124–132), as well as the conformational flexibility of a long extended loop where the epitopes 110–118, and the more variable 124–132 are located. Our method provides a means to identify linear epitopes which bind to antibodies with desirable characteristics for virus neutralization.

The method is based on sequence information only but the prediction accuracy is improved when 3D structural data of the antigen (and or antibody) is known. The method is based on an intuitive concept that does not critically depend on the knowledge of the exact fold of CDR loop regions as we have shown previously [2,3]. Our method might, therefore, also hint to a possible expanded understanding of antibody-antigen binding determinants. While the predicted epitopes still need to be experimentally verified, the amount of experimental work is significantly reduced and focused on well specified sequence regions. The predicted and verified epitopes can then be used in vaccine design to elicit a neutralizing antibody response. Conversely, the method can also be used to identify epitopes which should be avoided such as those that elicit AER.

## Figures and Tables

**Table 1:** Distribution of key minima and the derived sequence pattern for the binding epitope peptides to antibody 1H7.4. A sequence pattern of “XZ” [X = R, H, Y, (Q or N) and Z = K, R, H, Y, (Q or N)] was obtained.

Binding Surface	S1	S2
**MCSS Minima Pattern**	MGUA	MGUA
	IMIA	MAMM
	PHEN	IMIA
	ACEM	PHEN
		ACEM
**Sequence Pattern**	R	R/K
	H	H
	Y	Y
	Q/N	Q/N

**Table 2:** Summary of the binder peptides of DENV2 for the antibody 1H7.4 predicted from sequence search only. The peptides which can be considered as potential epitopes are highlighted in bold.

Peptides	Regions	Sequence	Length (mer)	Secondary Structure	Surface Accessibility	Epitopes
1H7.4-P1	5–13	vvswknkel	9	Turn	Exposed ^a^	
**1H7.4-P2**	27–35	twteqykfq	9	Loop	exposed	**1**
1H7.4-P3	65–80	nlmwkqitpelnhils	16	Helix	exposed	
**1H7.4-P4**	97–116	mqagkrslrpqptelkyswk	20	Loop	exposed	**2**
**1H7.4-P5**	125–134	steshnqtfl	10	Loop	exposed	**3**
**1H7.4-P6**	143–151	cpntnrawn	9	Loop	exposed	**4**
**1H7.4-P7**	170–178	klrekqdvf	9	Loop	exposed	**5**
1H7.4-P8	187–195	aikdnravh	9	2β stands	buried	
1H7.4-P9	249–264	gpvsqhnyrpgyytqt	16	Loop	dimer interface ^b^	
**1H7.4-P10**	289–297	edcgnrgps	9	Loop	exposed	**6**
1H7.4-P11	318–327	lpplryrged	10	β stand	buried	

^a^ peptide is exposed and close to membrane surface (see Figure S1 in Supplementary Materials); ^b^ peptide is located on the dimer interface (see Figure S1 in Supplementary Materials).

**Table 3:** Distribution of key minima and the derived sequence pattern for the binding epitope peptides to the antibody 1G5.3. A sequence pattern of “X–Z” [X = (I or L or V), Y, W and Z = Y, W, (D or E)] was obtained.

Binding Site	B1	11.50 Å	B2
**MCSS minima Pattern**	IBUT		PHEN
	PHEN		INDO
	INDO		ACET
**Sequence Pattern**	I/L/V	Gap of 2 amino acid	Y
	Y		W
	W		D/E

**Table 4:** Summary of the binder peptides of DENV2 for the antibody 1G5.3, predicted from sequence search only. The peptides that can be considered as potential epitopes are highlighted in bold.

Peptides	Regions	Sequence	Length (mer)	Secondary Structure	Surface Accessibility	Epitopes
1G5.3-P1	3–9	gcvvswk	7	β stand	exposed a	
1G5.3-P2	21–29	itdnvhtwt	9	loop	buried	
1G5.3-P3	67–82	mwkqitpelnhilsen	16	helix	exposed	
**1G5.3-P4**	111–128	lkyswktwgkakmlstes	18	loop	exposed	**I**
1G5.3-P5	149–159	awnslevedyg	11	β stand	buried	
1G5.3-P6	173–181	ekqdvfcds	9	loop	buried b	
1G5.3-P7	190–214	dnravhadmgywiesalndtwkmek	25	2β stands	buried	
**1G5.3-P8**	233–241	sngvlesem	9	loop	exposed	**II**
1G5.3-P9	269–277	hlgklemdf	9	β stand	buried	
**1G5.3-P10**	282–291	gttvvvtedc	10	loop	exposed	**III**
**1G5.3-P11**	303–312	asgklitewc	10	loop	exposed	**IV**
1G5.3-P12	319–335	pplryrgedgcwygmei	17	2 β stands	buried	

^a^ peptide is exposed and close to membrane surface (see Figure S2 in Supplementary Materials); ^b^ peptide is buried in the dimer form (see Figure S2 in Supplementary Materials).

**Table 5:** Distribution of key minima and the derived sequence pattern for the binding epitope peptides to the antibody GUS2. A sequence pattern of “XZ” [X = Y, (Q or N), (R or K) and Z = F, H, Y, W, (Q or N), (R or K)] was obtained.

Binding Site	C1	C2
**MCSS Minima Pattern**	PHEN	BENZ
	ACEM	IMIA
	MAMM	PHEN
	MGUA	INDO
		ACEM
		MAMM
		MGUA
**Sequence Pattern**	Y	F
	Q/N	H
	R/K	Y
		W
		Q/N
		R/K

**Table 6:** Summary of the binder peptides of DENV2 for the antibody GUS2, predicted from sequence search only. The peptides that can be considered as potential epitopes are highlighted in bold.

Peptides	Regions	Sequence	Length (mer)	Secondary Structure	Surface Accessibility	Epitopes
GUS2-P1	3–13	gcvvswknkel	11	2 β stands	exposed ^a^	
**GUS2-P2**	27–36	twteqykfqp	10	loop	exposed	**A**
GUS2-P3	42–51	lasaiqkahe	10	helix	buried	
GUS2-P4	63–71	lenlmwkqi	9	helix	exposed	
**GUS2-P5**	110–118	elkyswktw	9	loop	exposed	**B**
**GUS2-P6**	124–132	lsteshnqt	9	loop	exposed	**C**
GUS2-P7	145–153	ntnrawnsl	9	loop	buried	
GUS2-P8	196–213	admgywiesalndtwkme	18	2 β stands	buried **^b^**	
**GUS2-P9**	249–264	gpvsqhnyrpgyytqt	16	loop	exposed	**D**
GUS2-P10	318–327	lpplryrged	10	β stand	buried	

^a^ peptide is exposed and close to membrane surface (see Figure S3 in Supplementary Materials); ^b^ peptide is located on the dimer interface (see Figure S3 in Supplementary Materials).

**Table 7:** The relationship between functional groups and amino acids.

	Functional Group	Abbreviation	Amino Acids
Charged (−)	Acetate ion	ACET	ASP, GLU
Charged (+)	Methylguanidinium	MGUA	ARG
Charged (+)	Methylammonium	MAMM	LYS
Polar	Acetamide	ACEM	ASN, GLN
Polar	Methanol	MEOH	SER, THR
Hydrophobic	Methanethiol	MESH	CYS, MET
Aromatic Polar	Phenol	PHEN	TYR
Aromatic Polar	Indole	INDO	TRP
Aromatic Polar	Imidazole	IMIA	HIS
Aromatic Hydrophobic	Benzene	BENZ	PHE
Hydrophobic	Ibutane	IBUT	VAL, ILE, LEU

## Supplementary Materials

Supplementary materials are available online.
