# Supplementary material for: Computational Identification of Antibody Epitopes on the Dengue Virus NS1 Protein
Source: Molecules. 2017 Apr 10;22(4):607. doi: 10.3390/molecules22040607 (PMC6154621; doi:10.3390/molecules22040607)
Supplement: Supplementary file 1 [file molecules-22-00607-s001.pdf]

Computational Identification of the Antibody Recognition Regions of Dengue NS1 proteins

Martina Jones[1], Fiona S. Legge[2], Kebaneilwe Lebani[1], Stephen Mahler[1], Paul Young[3], Daniel Watterson[3], Herbert R. Treutlein[2,4*] and Jun Zeng[2,4]*

1. Australian Institute for Bioengineering and Nanotechnology, The University of Queensland, St Lucia, QLD 4067, Australia
2. Computist Bio-Nanotech, 1 Dalmore Drive, Scoresby, VIC 3179, Australia
3. School of Chemistry and Molecular Biosciences, The University of Queensland, St Lucia, QLD 4067, Australia
4. School of Medical Sciences, RMIT University, PO Box 71, Bundoora, Victoria 3083, Australia

*Corresponding authors.

Herbert Treutlein: +61 412 367 935, email: Herbert.Treutlein@computistresearch.com, Jun Zeng: +61 4 13323321, Email: Jun.Zeng@computistresearch.com,

Supplementary Materials

A)

DSGCvvswknkelKCGSGIFITDNVHtwteqykfqPESPSKLASAIQKAHEEGICGIRSVTRLEnlmwk qitpelnhilsENEVKLTIMTGDIKGImqagkrslrpqptelkyswkTWGKAKMLsteshnqtflI DGPETAEcpntnrawnSLEVEDYGFGVFTTNIWLklrekqdvfCDSKLMSAaikdnravhADMGYW IESALNDTWKMEKASFIEVKSCHWPKSHTLWSNGVLESEMIIPKSFAgpvsqhnyrpgyytqtAGP WHLGKLEMDFDFCEGTTVVVTedcgnrgpsLRTTTASGKLITEWCCRSCTlpplryrgedGCWYGM EIRPLKEKEENLVNSLVTA

B)

Summary of the binder peptides (as listed above) of DENV2 for the antibody 1H7.4 predicted from sequence search only. The peptides can be considered as epitopes are highlighted in bold and colored in magenta.

**Peptides**	**Regions**	**Sequence**	**Length (mer)**	**Secondary Structure**	**Surface Accessibility**	**Epitopes**
1H7.4-P1	5-13	vvswknkel	9	Turn	Exposed [a]	
**1H7.4-P2**	**27-35**	**twteqykfq**	**9**	**Loop**	**exposed**	**1**
1H7.4-P3	65-80	nlmwkqitpelnhils	16	Helix	exposed	
**1H7.4-P4**	**97-116**	**mqagkrslrpqptelkyswk**	**19**	**Loop**	**exposed**	**2**
**1H7.4-P5**	**125-133**	**steshnqtfl**	**10**	**Loop**	**exposed**	**3**
**1H7.4-P6**	**143-151**	**cpntnrawn**	**9**	**Loop**	**exposed**	**4**
**1H7.4-P7**	**170-178**	**klrekqdvf**	**9**	**Loop**	**exposed**	**5**
1H7.4-P8	187-195	aikdnravh	9	2β stands	buried	
1H7.4-P9	249-264	gpvsqhnyrpgyytqt	16	Loop	dimer interface [b]	
**1H7.4-P10**	**289-297**	**edcgnrgps**	**9**	**Loop**	**exposed**	**6**
1H7.4-P11	318-327	lpplryrged	10	β stand	buried	

[a] pepide is exposed and close to membrane surface.

[b] peptide is located on the dimer interface.

C)

D)

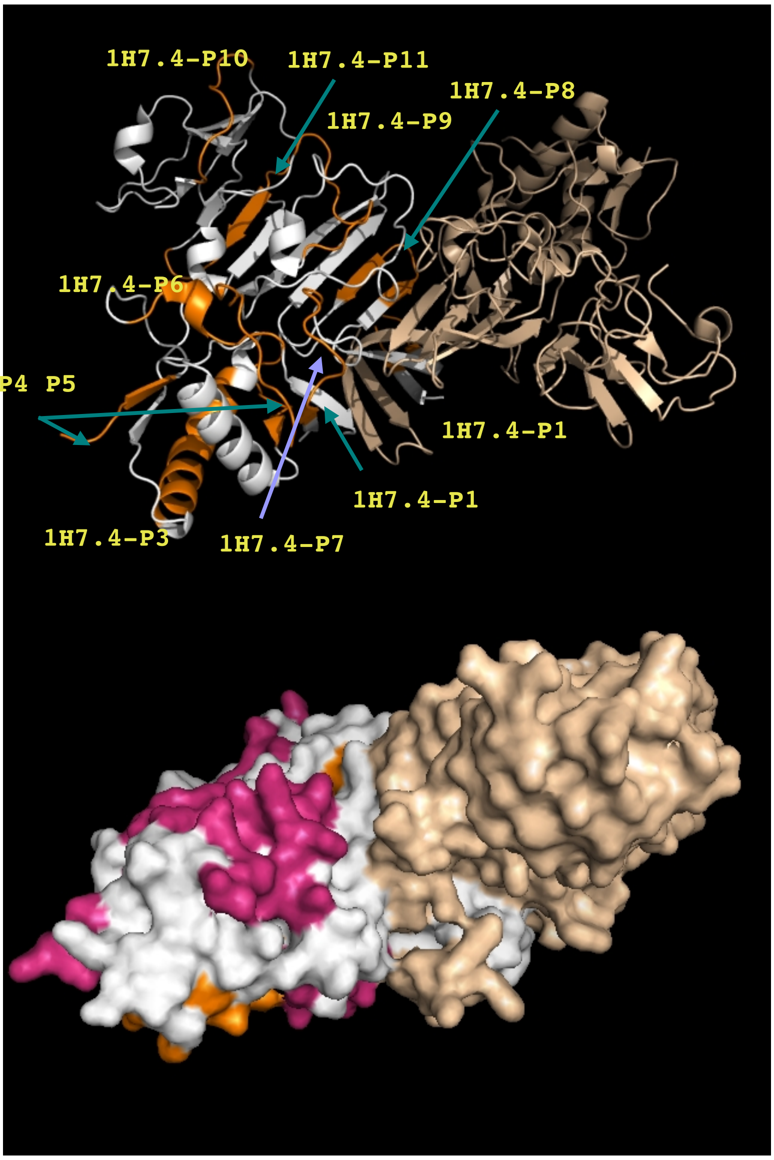

Figure S1: Predicted Binder peptides of DENV2 to the antibody 1H7.4. A) the sequence of DENV2 NS1 protein with the binder peptides highlighted in lower case and colored in orange. The final epitopes are colored in magenta. B) Summary of the binder peptides according to the secondary structure and surface accessibility. C) Illustration of the binder peptides in the crystal structure of dimer form of DENV2 NS1 protein. D) the location of the binder peptides on the surface of dimer form of DENV2 NS1 protein. The final epitopes selected based on secondary structure and surface accessibility are colored in magenta.

A)

DSgcvvswkNKELKCGSGIFitdnvhtwtEQYKFQPESPSKLASAIQKAHEEGICGIRSVTRLENL
mwkqitpelnhilsenEVKLTIMTGDIKGIMQAGKRSLRPQPTElkyswktwgkakmlstesHNQT
FLIDGPETAECPNTNRawnslevedygFGVFTTNIWLKLRekqdvfcdsKLMSAAIKdnravhadm
gywiesalndtwkmekASFIEVKSCHWPKSHTLWsngvlesemIIPKSFAGPVSQHNYRPGYYTQT
AGPWhlgklemdfDFCEgttvvvtedcGNRGPSLRTTTasgklitewcCRSCTLpplryrgedgcw
ygmeiRPLKEKEENLVNSLVTA

B)

Summary of the binder peptides of DENV2 (shown in above) for the antibody 1G5.3 predicted from sequence search only. The peptides can be considered as epitopes are highlighted in bold and colored in magenta.

Peptides	Regions	Sequence	Length (mer)	Secondary Structure	Surface Accessibility	Epitopes
1G5.3-P1	2-9	gcvvswk	7	β stand	exposed [a]	
1G5.3-P2	21-29	itdnvhtwt	9	loop	buried	
1G5.3-P3	67-82	mwkqitpelnhilsen	16	helix	exposed	
**1G5.3-P4**	**111-128**	**lkyswktwgkakmlstes**	**18**	**loop**	**exposed**	**I**
1G5.3-P5	149-159	awnslevedyg	10	β stand	buried	
1G5.3-P6	173-181	ekqdvfcds	9	loop	buried [b]	
1G5.3-P7	190-214	dnravhadmgywiesalndtw kmek	24	2 β stands	buried	
**1G5.3-P8**	**233-241**	**sngvlesem**	**9**	**loop**	**exposed**	**II**
1G5.3-P9	269-277	hlgklemdf	9	β stand	buried	
**1G5.3-P10**	**282-291**	**gttvvvtedc**	**10**	**loop**	**exposed**	**III**
**1G5.3-P11**	**303-312**	**asgklitewc**	**10**	**loop**	**exposed**	**IV**
1G5.3-P12	319-335	pplryrgedgcwygmei	17	2 β stands	buried	

[a] pepide is exposed and close to membrane surface (Panel B).

[b] peptide is buried in the dimer form (Panel B)

C)

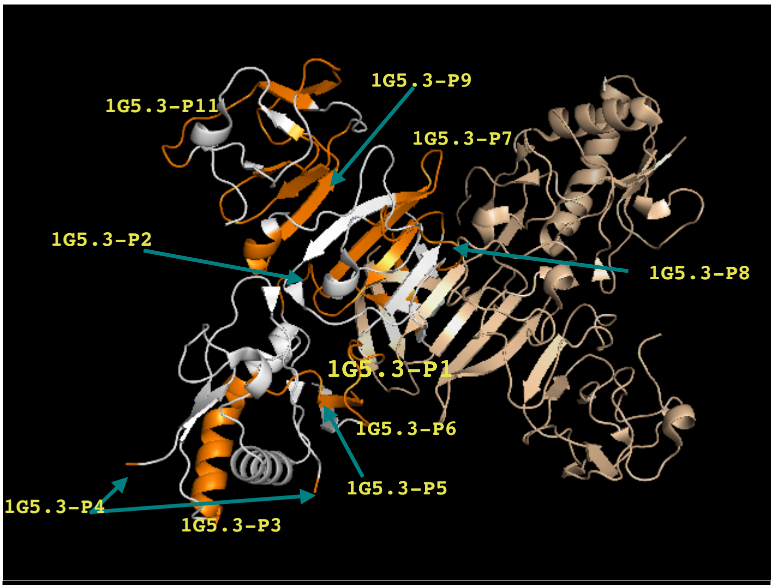

D)

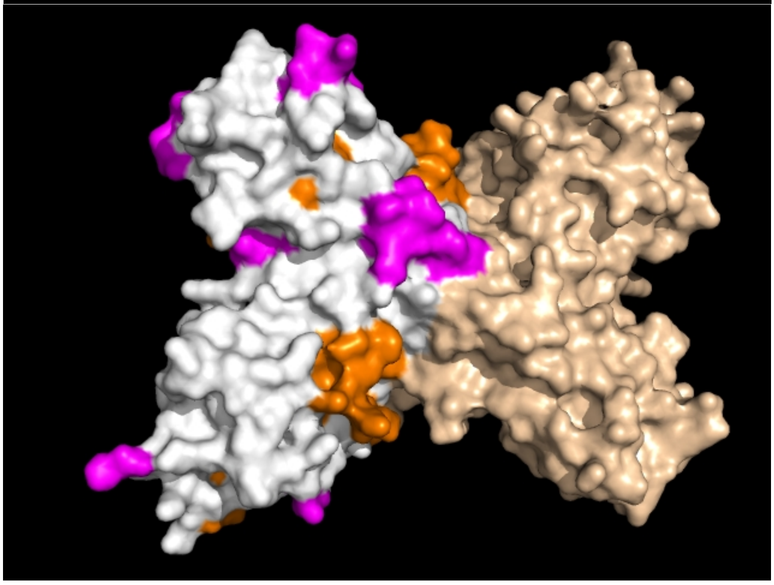

Figure S2: Predicted Binder peptides of DENV2 to the antibody 1G5.3. A) the sequence of DENV2 NS1 protein with the binder peptides highlighted in lower case and colored in orange. The final epitopes are colored in magenta. B) Summary of the binder peptides according to the secondary structure and surface accessibility. C) Illustration of the binder peptides in the crystal structure of dimer form of DENV2 NS1 protein. D) the location of the binder peptides on the surface of dimer form of DENV2 NS1 protein. The final epitopes selected based on secondary structure and surface accessibility are colored in magenta.

A)

DSgcvvswknkelKCGSGIFITDNVHtwteqykfqpESPSKlasaiqkaheEGICGIRSVTRlenl
mwkqiTPELNHILSENEVKLTIMTGDIKGIMQAGKRSLRPQPTelkyswktwGKAKMlsteshnqt
FLIDGPETAECPntnrawnslEVEDYGFGVFTTNIWLKLREKQDVFCDSKLMSAAIKDNRAVHadm
gywiesalndtwkmeKASFIEVKSCHWPKSHTLWSNGVLESEMIIPKSFAgpvsqhnyrpgyytqt
AGPWHLGKLEMDFDFCEGTTVVVTEDCGNRGPSLRTTTASGKLITEWCCRSCTlpplryrgedGCW
YGMEIRPLKEKEENLVNSLVTA

B)

Summary of the binder peptides of DENV2 (shown in above) for the antibody GUS2 predicted from sequence search only. The peptides can be considered as epitopes are highlighted in bold and colored in magenta.

Peptides	Regions	Sequence	Length (mer)	Secondary Structure	Surface Accessibility	Epitopes
GUS2-P1	3-13	gcvvswknkel	11	2 β stands	exposed [a]	
**GUS2-P2**	**27-36**	**twteqykfqp**	**10**	**loop**	**exposed**	**A**
GUS2-P3	42-51	lasaiqkahe	10	helix	buried	
GUS2-P4	63-71	lenlmwkqi	9	helix	exposed	
**GUS2-P5**	**110-118**	**elkyswktw**	**9**	**loop**	**exposed**	**B**
**GUS2-P6**	**124-132**	**lsteshnqt**	**9**	**loop**	**exposed**	**C**
GUS2-P7	145-153	ntnrawnsl	9	loop	buried	
GUS2-P8	196-213	admgywiesalndtwkme	18	2 β stands	Buried [b]	
**GUS2-P9**	**249-264**	**gpvsqhnyrpgyytqt**	**16**	**loop**	**exposed**	**D**
GUS2-P10	318-327	lpplryrged	10	β stand	buried	

[a] pepide is exposed and close to membrane surface (Panel B).

[b] peptide is located on the dimer interface (Panel B)

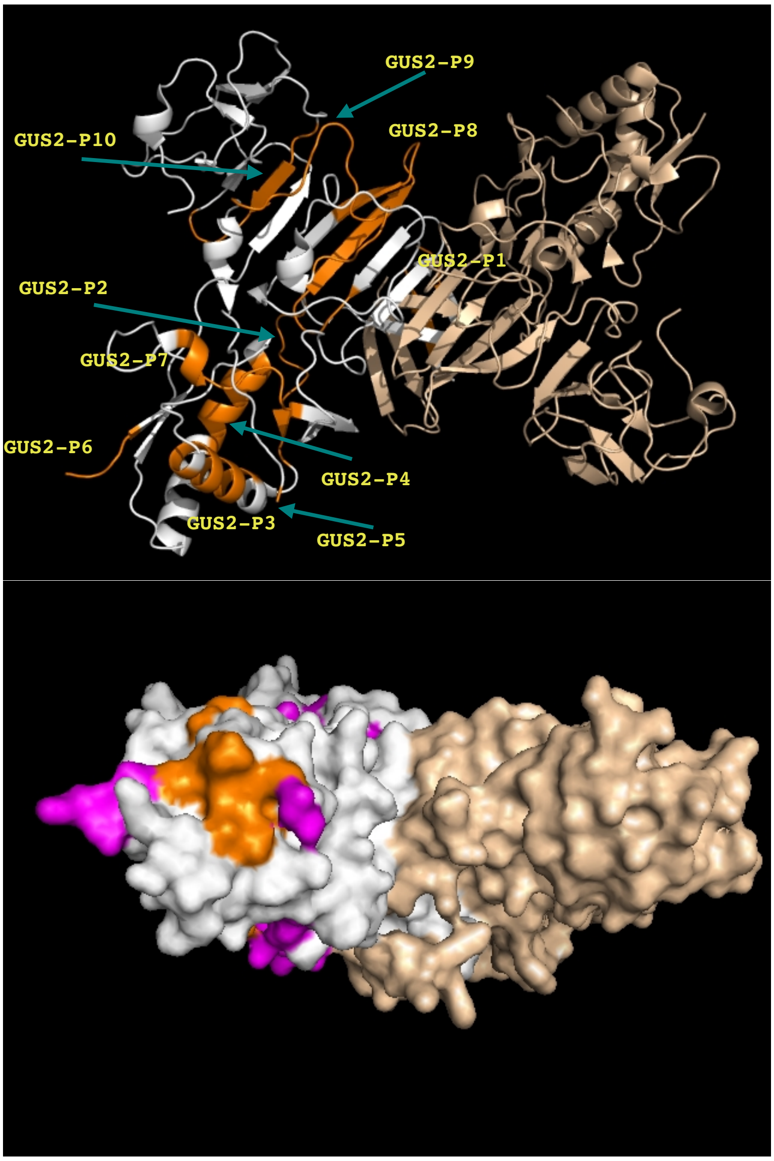

Figure S3: Predicted Binder peptides of DENV2 to the antibody GUS2. A) the sequence of DENV2 NS1 protein with the binder peptides highlighted in lower case and colored in orange. The final epitopes are colored in magenta. B) Summary of the binder peptides according to the secondary structure and surface accessibility. C) Illustration of the binder peptides in the crystal structure of dimer form of DENV2 NS1 protein. D) the location of the binder peptides on the surface of dimer form of DENV2 NS1 protein. The final epitopes selected based on secondary structure and surface accessibility are colored in magenta.

Appendix S1: peptide libraries of NS1 proteins of DENV serotypes bind to antibody 1H7.4

DENV1

1 DSGCVINWKGRELKCGSGIFVTNEVHTWteqykfqadspkrlSAAIGKAWEEGVCGIRSATRLenimwkqisnelnhILLENDMKFTVVVGDVSGILA
2 DSGCVINWKGRELKCGSGIFVTNEVHTWTeqykfqadspkrlsAAIGKAWEEGVCGIRSATRLEnimwkqisnelnhiLLENDMKFTVVVGDVSGILA
3 DSGCVINWKGRELKCGSGIFVTNEVHTWTEqykfqadspkrlsaAIGKAWEEGVCGIRSATRLENimwkqisnelnhilLENDMKFTVVVGDVSGILA
4 DSGCVINWKGRELKCGSGIFVTNEvhtwteqYKFQADSpkrlsaaIGKAWEEGVCGIRSATRLENImwkqisnelnhillENDMKFTVVVGDVSGILA
5 DSGCVINWKGRELKCGSGIFVTNEVhtwteqyKFQADSPkrlsaaiGKAWEEGVCGIRSATRLENIMwkqisnelnhilleNDMKFTVVVGDVSGILA
6 DSGCVINWKGRELKCGSGIFVTNEVHtwteqykfqadspkRLSAAIGKAWEEGVCGIRSATRLENIMWkqisnelnhillenDMKFTVVVGDVSGILA
7 DSGCVINWKGRELKCGSGIFVTNEVHTwteqykfqadspkrLSAAIGKAWEEGVCGIRSATRlenimwkqisnelnHILLENDMKFTVVVGDVSGILA

1 QGKKMIRpqpmehkYSWKSWGKAKIIGAdvqnttfIIDGPNTpecpdnqRAWNIWEVEDYGFGIFTTNIWLKLRDSYtqvcdhrLMSAAIKDSKAVHA
2 QGKKMIRPqpmehkySWKSWGKAKIIGADvqnttfiIDGPNTPecpdnqrAWNIWEVEDYGFGIFTTNIWLKLRDSYTqvcdhrlMSAAIKDSKAVHA
3 QGKKMIRPQpmehkysWKSWGKAKIIGADVqnttfiiDGPNTPEcpdnqraWNIWEVEDYGFGIFTTNIWLKLRDSYTQvcdhrlmSAAIKDSKAVHA
4 QGKKMIRPQPmehkyswKSWGKAKiigadvqNTTFIIDGPNTPECpdnqrawNIWEVEDYGFGIFTTNIWLKLRDSYTQVcdhrlmsAAIKDSKAVHA
5 QGKKMIRPQPMehkyswkSWGKAKIigadvqnTTFIIDGPNTPECPdnqrawnIWEVEDYGFGIFTTNIWLKLRDSYTQVCdhrlmsaAIKDSKAVHA
6 QGKKMIRPQPMEhkyswksWGKAKIIgadvqntTFIIDGPNTPECPDnqrawniWEVEDYGFGIFTTNIWLKLRDSYTQVCDhrlmsaaIKDSKAVHA
7 QGKKMIRPQPMEHkyswkswGKAKIIGadvqnttFIIDGPNtpecpdnqrawniwEVEDYGFGIFTTNIWLKLRDSytqvcdhRLMSAAIKDSKAVHA

1 DMGYWIEseknetwKLARASFIEVKTCIWPKSHTLWSNGVLESEMIIPKIYGGPISqhnyrpgYFTQTAGPWHLGKLELDFDLCEGTTVVVdehcgnr
2 DMGYWIESeknetwkLARASFIEVKTCIWPKSHTLWSNGVLESEMIIPKIyggpisqhnyrpgyFTQTAGPWHLGKLELDFDLCEGTTVVVDehcgnr
3 DMGYWIESEknetwklARASFIEVKTCIWPKSHTLWSNGVLESEMIIPKIYggpisqhnyrpgyfTQTAGPWHLGKLELDFDLCEGTTVVVDEhcgnr
4 DMGywiesekNETWKLARASFIEVKTCIWPKSHTLWSNGVLESEMIIPKIYGgpisqhnyrpgyftQTAGPWHLGKLELDFDLCEGTTVVVDEHcgnr
5 DMGYwieseknETWKLARASFIEVKTCIWPKSHTLWSNGVLESEMIIPKIYGGpisqhnyRPGYFTQTAGPWHLGKLELDFDLCEGTTVVVDEHCgnr
6 DMGYWieskneTWKLARASFIEVKTCIWPKSHTLWSNGVLESEMIIPKIYGGPisqhnyrPGYFTQTAGPWHLGKLELDFDLCEGTTVVVDEHCGnr
7 DMGYWIeseknetWKLARASFIEVKTCIWPKSHTLWSNGVLESEMIIPKIYGGPIsqhnyrpGYFTQTAGPWHLGKLELDFDLCEGTTVVvdehcgnR

1 GPSLRTTTVTGKTIHEWCCRSCTLPPLRFKGEDGCWYGMEIRPVKEKEENLVKSMVSA
2 gPSLRTTTVTGKTIHEWCCRSCTLPPLRFKGEDGCWYGMEIRPVKEKEENLVKSMVSA
3 gpSLRTTTVTGKTIHEWCCRSCTLPPLRFKGEDGCWYGMEIRPVKEKEENLVKSMVSA
4 gpsLRTTTVTGKTIHEWCCRSCTLPPLRFKGEDGCWYGMEIRPVKEKEENLVKSMVSA
5 gpslRTTTVTGKTIHEWCCRSCTLPPLRFKGEDGCWYGMEIRPVKEKEENLVKSMVSA
6 gpslrTTTVTGKTIHEWCCRSCTLPPLRFKGEDGCWYGMEIRPVKEKEENLVKSMVSA
7 GPSLRTTTVTGKTIHEWCCRSCTLPPLRFKGEDGCWYGMEIRPVKEKEENLVKSMVSA

DENV2

1 DSGCVVSwknkelkCGSGIFITDNVHTWteqykfqPESPSKLASAIQKAHEEGICGIRSVTRLenlmwkqitpelnhILSENEVKLTIMTGDIKGIMQ
2 DSGCVVSWknkelkcGSGIFITDNVHTWTeqykfqpESPSKLASAIQKAHEEGICGIRSVTRLEnlmwkqitpelnhiLSENEVKLTIMTGDIKGIMQ
3 DsgcvvswkNKELKCGSGIFITDNVHTWTEqykfqpeSPSKLASAIQKAHEEGICGIRSVTRLENlmwkqitpelnhilSENEVKLTIMTGDIKGIMQ
4 DSGcvvswknKELKCGSGIFITDNvhtwteqYKFQPESPSKLASAIQKAHEEGICGIRSVTRLENLmwkqitpelnhilsENEVKLTIMTGDIKgimq
5 DSGCvvswknkELKCGSGIFITDNVhtwteqyKFQPESPSKLASAIQKAHEEGICGIRSVTRLENLMwkqitpelnhilseNEVKLTIMTGDIKGimq
6 DSGCVvswknkeLKCGSGIFITDNVHtwteqykFQPESPSKLASAIQKAHEEGICGIRSVTRLENLMWkqitpelnhilsenEVKLTIMTGDIKGImq
7 DSGCVVswknkelKCGSGIFITDNVHTwteqykfQPESPSKLASAIQKAHEEGICGIRSVTRlenlmwkqitpelnHILSENEVKLTIMTGDIKGIMq

1 agkrslrpqptelkYSWKTWGKAKMLSTeshnqtfLIDGPETaecpntnRAWNSLEVEDYGFGVFTTNIWlklrekqDVFCDSKLMSAAIKdnravha
2 AgkrslrpqptelkySWKTWGKAKMLSTEshnqtflIDGPETAecpntnrAWNSLEVEDYGFGVFTTNIWLklrekqdVFCDSKLMSAAIKDnravha
3 AGkrslrpqptelkysWKTWGKAKMLSTEShnqtfliDGPETAEcpntnraWNSLEVEDYGFGVFTTNIWLKlrekqdvFCDSKLMsaaikdnRAVHA
4 agkRSLRPQPtelkyswKTWGKAKmlsteshnqtflidGPETAECpntnrawNSLEVEDYGFGVFTTNIWLKLrekqdvfCDSKLMSaaikdnrAVHA
5 agkrSLRPQPTelkyswkTWGKAKMlsteshnQTFLIDGPETAECPntnrawnSLEVEDYGFGVFTTNIWLKLRekqdvfcDSKLMSAaikdnraVHA
6 agkrsLRPQPTElkyswktWGKAKMLsteshnqTFLIDGPETAECPNtnrawnsLEVEDYGFGVFTTNIWLKLREkqdvfcdSKLMSAAikdnravHA
7 agkrslRPQPTELkyswktwGKAKMLSteshnqtFLIDGPETAECPNTnrawnslEVEDYGFGVFTTNIwlklrekQDVFCDSKLMSAAIkdnravhA

1 DMGYWIESALNDTWKMEKASFIEVKSCHWPKSHTLWSNGVLESEMIIPKSFAGPVSqhnyrpgyytqtagPWHLGKLEMDFDFCEGTTVVVtedcgnr
2 dMGYWIESALNDTWKMEKASFIEVKSCHWPKSHTLWSNGVLESEMIIPKSfagpvsqhnyrpgyYTQTAGPWHLGKLEMDFDFCEGTTVVVTedcgnr
3 DMGYWIESALNDTWKMEKASFIEVKSCHWPKSHTLWSNGVLESEMIIPKSFagpvsqhnyrpgyyTQTAGPWHLGKLEMDFDFCEGTTVVVTEdcgnr
4 DMGYWIESALNDTWKMEKASFIEVKSCHWPKSHTLWSNGVLESEMIIPKSFAgpvsqhnyrpgyytQTAGPWHLGKLEMDFDFCEGTTVVVTEDcgnr
5 DMGYWIESALNDTWKMEKASFIEVKSCHWPKSHTLWSNGVLESEMIIPKSFAGpvsqhnyrpgyytqTAGPWHLGKLEMDFDFCEGTTVVVTEDCgnr
6 DMGYWIESALNDTWKMEKASFIEVKSCHWPKSHTLWSNGVLESEMIIPKSFAGPvsqhnyrpgyytqtAGPWHLGKLEMDFDFCEGTTVVVTEDCGnr
7 DMGYWIESALNDTWKMEKASFIEVKSCHWPKSHTLWSNGVLESEMIIPKSFAGPVsqhnyrpgyytqtaGPWHLGKLEMDFDFCEGTTVVvtedcgnR

1 GPSLRTTTASGKLITEWCCRSctlpplryrgedgcWYGMEIRPLKEKEENLVNSLVTA
2 gPSLRTTTASGKLITEWCCRSCtlpplryRGEDGCWYGMEIRPLKEKEENLVNSLVTA
3 gpSLRTTTASGKLITEWCCRSCTlpplryrGEDGCWYGMEIRPLKEKEENLVNSLVTA
4 gpsLRTTTASGKLITEWCCRSCTLpplryrgEDGCWYGMEIRPLKEKEENLVNSLVTA
5 gpslRTTTASGKLITEWCCRSCTLPplryrgeDGCWYGMEIRPLKEKEENLVNSLVTA
6 gpslrTTTASGKLITEWCCRSCTLPPlryrgedGCWYGMEIRPLKEKEENLVNSLVTA
7 GPSLRTTTASGKLITEWCCRSCTLPPLryrgedgCWYGMEIRPLKEKEENLVNSLVTA

DENV3

1 DMGCVINWKGKELKCGSGIFVTNEVHTWteqykfqadspkrlATAIAGAWENGVCGIRSTTRMenllwkqianelnyilwenniKLTVVVGDITGVLE
2 DMGCVINWKGKELKCGSGIFVTNEVHTWTeqykfqadspkrlaTAIAGAWENGVCGIRSTTRMEnllwkqianelnyilwennikLTVVVGDITGVLE
3 DMGCVINWKGKELKCGSGIFVTNEVHTWTEqykfqadspkrlatAIAGAWENGVCGIRSTTRMENllwkqianelnyilwenniklTVVVGDITGVLE
4 DMGCVINWKGKELKCGSGIFVTNEvhtwteqYKFQADSpkrlataIAGAWENGVCGIRSTTRMENLlwkqianelnyilwenniklτVVVGDITgvle
5 DMGCVINWKGKELKCGSGIFVTNEVhtwteqyKFQADSPkrlataiAGAWENGVCGIRSTTRMENLLwkqianelnyilwenniklt vVVGDITGvle
6 DMGCVINWKGKELKCGSGIFVTNEVHtwteqykfqadspkRLATAIAGAWENGVCGIRSTTRMENLLWkqianelnyilwenNIKLTVVVGDITGVle
7 DMGCVINWKGKELKCGSGIFVTNEVHTwteqykfqadspkrLATAIAGAWENGVCGIRSTTRmenllwkqianelnyilwennIKLTVVVGDITGVLe

1 qgkrtltpqpmelkYSWKTWGKAKIVTAetqnssfIIDGPSTPECPSASRAWNVWEVEDYGFGVFTTNIWLKLREVYtqlcdhrLMSAAVKDERAVHA
2 QgkrtltpqpmelkySWKTWGKAKIVTAEtqnssfiIDGPSTPECPSASRAWNVWEVEDYGFGVFTTNIWLKLREVYTqlcdhrlMSAAVKDERAVHA
3 QQkrtltpqpmelkysWKTWGKAKIVTAETqnssfiiDGPSTPECPSASRAWNVWEVEDYGFGVFTTNIWLKLREVYTQlcdhrlmSAAVKDERAVHA
4 qgkRTLTPQPmelkyswKTWGKAKivtaetqNSSFIIDGPSTPECPSASRAWNVWEVEDYGFGVFTTNIWLKLREVYTQLcdhrlmsAAVKDERAVHA
5 qgkrTLTPQPMelkyswkTWGKAKIvtaetqnSSFIIDGPSTPECPSASRAWNVWEVEDYGFGVFTTNIWLKLREVYTQLCdhrlmsaAVKDERAVHA
6 qgkrtLTPQPMElkyswktWGKAKIVtaetqnsSFIIDGPSTPECPSASRAWNVWEVEDYGFGVFTTNIWLKLREVYTQLCDhrlmsaaVKDERAVHA
7 qgkrtlTPQPMELkyswktwGKAKIVTaetqnssFIIDGPSTPECPSASRAWNVWEVEDYGFGVFTTNIWLKLREVytqlcdhRLMSAAVKDERAVHA

1 DMGYWIEsqkngswKLEKASLIEVKTCTWPKSHTLWSNGVLESDMIIPKSLAGPISqhnhrpgyhtqtagPWHLGKLeldfnycEGTTVVISENCGTR
2 DMGYWIESqkngswkLEKASLIEVKTCTWPKSHTLWSNGVLESDMIIPKSlagpisqhnhrpgyHTQTAGPWHLGKLEldfnyceGTTVVISENCGTR
3 DMGYWIESQkngswklEKASLIEVKTCTWPKSHTLWSNGVLESDMIIPKSLagpisqhnhrpgyhTQTAGPWHLGKLELdfnycegTTVVISENCGTR
4 DMGywiesqkNGSWKLEKASLIEVKTCTWPKSHTLWSNGVLESDMIIPKSLAgpisqhnhrpgyhtQTAGPWHLGKLELDfnycegtTVVISENCGTR
5 DMGYwiesqknGSWKLEKASLIEVKTCTWPKSHTLWSNGVLESDMIIPKSLAGpisqhnhrpgyhtqTAGPWHLGKLELDFnycegttVVISENCGTR
6 DMGYWiesqkngSWKLEKASLIEVKTCTWPKSHTLWSNGVLESDMIIPKSLAGPisqhnhrpgyhtqtAGPWHLGkleldfnYCEGTTVVISENCGTR
7 DMGYWIesqkngsWKLEKASLIEVKTCTWPKSHTLWSNGVLESDMIIPKSLAGPIsqhnhrpgyhtqtaGPWHLGKleldfnyCEGTTVVISENCGTR

1 GPSLRTTTVSGKLIHEWCCRSctlpplrYMGEDGCWYGMEIRPINEKEENMVKSLASA
2 GPSLRTTTVSGKLIHEWCCRSCtlpplryMGEDGCWYGMEIRPINEKEENMVKSLASA
3 GPSLRTTTVSGKLIHEWCCRSCTlpplrymGEDGCWYGMEIRPINEKEENMVKSLASA
4 GPSLRTTTVSGKLIHEWCCRSCTLpplrymgEDGCWYGMEIRPINEKEENMVKSLASA
5 GPSLRTTTVSGKLIHEWCCRSCTLPplrymgeDGCWYGMEIRPINEKEENMVKSLASA
6 GPSLRTTTVSGKLIHEWCCRSCTLPPlrymgedGCWYGMEIRPINEKEENMVKSLASA
7 GPSLRTTTVSGKLIHEWCCRSCTLPPLrymgedgCWYGMEIRPINEKEENMVKSLASA

DENV4

1 DMGCVVSWSGKELKCGSGIFVADNVHTWteqykfqPESPARLASAILNAHKDGVCGIRSTTRLenvmwkqitnelnyVLWEGGHDLTVVAGDVKGVLT
2 DMGCVVSWSGKELKCGSGIFVADNVHTWTeqykfqpESPARLASAILNAHKDGVCGIRSTTRLEnvmwkqitnelnyvLWEGGHDLTVVAGDVKGVLT
3 DMGCVVSWSGKELKCGSGIFVADNVHTWTEqykfqpeSPARLASAILNAHKDGVCGIRSTTRLENvmwkqitnelnyvlWEGGHDLTVVAGDVKGVLT
4 DMGCVVSWSGKELKCGSGIFVADNvhtwteqYKFQPESPARLASAILNAHKDGVCGIRSTTRLENVmwkqitnelnyvlwEGGHDLTVVAGDVKgvlt
5 DMGCVVSWSGKELKCGSGIFVADNVhtwteqyKFQPESPARLASAILNAHKDGVCGIRSTTRLENVMwkqitnelnyvlweGGHDLTVVAGDVKGvlt
6 DMGCVVSWSGKELKCGSGIFVADNVHtwteqykFQPESPARLASAILNAHKDGVCGIRSTTRLENVMWkqitnelnyvlwegGHDLTVVAGDVKGVlt
7 DMGCVVSWSGKELKCGSGIFVADNVHTwteqykfQPESPARLASAILNAHKDGVCGIRSTTRlenvmwkqitnelnYVLWEGGHDLTVVAGDVKGVLt

1 kgkraltppvndlkYSWKTWGKAKIFTPearnstfLIDGPDTsecpnerRAWNFLEVEDYGFGMFTTNIWMKFREGSsevcdhrLMSAAIKDQKAVHA
2 KgkraltppvndlkySWKTWGKAKIFTPEarnstflIDGPDTSecpnerrAWNFLEVEDYGFGMFTTNIWMKFREGSSevcdhrlMSAAIKDQKAVHA
3 KGkraltppvndlkysWKTWGKAKIFTPEArnstfliDGPDTSEcpnerraWNFLEVEDYGFGMFTTNIWMKFREGSSEvcdhrlmSAAIKDQKAVHA
4 kgkRALTPPVndlkyswKTWGKAKiftpearNSTFLIDGPDTSECpnerrawNFLEVEDYGFGMFTTNIWMKFREGSSEVcdhrlmsAAIKDQKAVHA
5 kgkrALTPPVNdlkyswkTWGKAKIftpearnSTFLIDGPDTSECPnerrawnFLEVEDYGFGMFTTNIWMKFREGSSEVCdhrlmsaAIKDQKAVHA
6 kgkraLTPPVNDlkyswktWGKAKIFtpearnsTFLIDGPDTSECPNerrawnfLEVEDYGFGMFTTNIWMKFREGSSEVCDhrlmsaaIKDQKAVHA
7 kgkralTPPVNDLkyswktwGKAKIFTpearnstFLIDGPDTSECPNErrawnflEVEDYGFGMFTTNIWMKFREGssevcdhRLMSAAIKDQKAVHA

1 DMGYWIEssknqtwQIEKASLIEVKTCLWPKTHTLWSNGVLESQMLIPKSYAGPFSqhnyrqgYATQTVGPWHLGKLEIDFGECPGTTVTIqedcdhr
2 DMGYWIESsknqtwqIEKASLIEVKTCLWPKTHTLWSNGVLESQMLIPKSyagpfsqhnyrqgyATQTVGPWHLGKLEIDFGECPGTTVTIQedcdhr
3 DMGYWIESSknqtwqiEKASLIEVKTCLWPKTHTLWSNGVLESQMLIPKSYagpfsqhnyrqgyaTQTVGPWHLGKLEIDFGECPGTTVTIQEdcdhr
4 DMGywiessknqtwqieKASLIEVKTCLWPKTHTLWSNGVLESQMLIPKSYAgpfsqhnyrqgyatQTVGPWHLGKLEIDFGECPGTTVTIQEDcdhr
5 DMGYwiessknQTWQIEKASLIEVKTCLWPKTHTLWSNGVLESQMLIPKSYAGpfsqhnyrqgyatqTVGPWHLGKLEIDFGECPGTTVTIQEDCdhr
6 DMGYWiessknqTWQIEKASLIEVKTCLWPKTHTLWSNGVLESQMLIPKSYAGPfsqhnyrQGYATQTVGPWHLGKLEIDFGECPGTTVTIQEDCDhr
7 DMGYWIessknqtWQIEKASLIEVKTCLWPKTHTLWSNGVLESQMLIPKSYAGPFsqhnyrqGYATQTVGPWHLGKLEIDFGECPGTTVTiqedcdhR

1 GPSLRTTTASGKLVTQWCCRSCTMPPLRFLGEDGCWYGMEIRPLSEKEENMVKSQVTA
2 gPSLRTTTASGKLVTQWCCRSCTMPPLRFLGEDGCWYGMEIRPLSEKEENMVKSQVTA
3 gpSLRTTTASGKLVTQWCCRSCTMPPLRFLGEDGCWYGMEIRPLSEKEENMVKSQVTA
4 gpsLRTTTASGKLVTQWCCRSCTMPPLRFLGEDGCWYGMEIRPLSEKEENMVKSQVTA
5 gpslRTTTASGKLVTQWCCRSCTMPPLRFLGEDGCWYGMEIRPLSEKEENMVKSQVTA
6 gpslrTTTASGKLVTQWCCRSCTMPPLRFLGEDGCWYGMEIRPLSEKEENMVKSQVTA
7 GPSLRTTTASGKLVTQWCCRSCTMPPLRFLGEDGCWYGMEIRPLSEKEENMVKSQVTA

Appendix S2: peptide libraries of NS1 proteins of DENV serotypes bind to antibody 1G5.3

DENV1

1 dsgcvinWKGRELKcgsgifvtnevhtwTEQYKFQADSPKRLSAAIGKAWEEGVCGIRSATRLENIMWKQisnelnhillendmkftvvvgDVSGILA
2 DsgcvinwKGRELKCgsgifvtnevhtwtEQYKFQADSPKRLSAAIGKAWEEGVCGIRSATRLEnimwkqisnelnhillendmkftvvvgdVSGILA
3 DSgcvinwkGRELKCGsgifvtnevhtwteQYKFQADSPKRLSAAIGKAWEEGVCGIRSATRLENimwkqisnelnhillendmkftvvvgdvSGILA
4 DSGcvinwkgRELKCGSgifvtnevhtwteqYKFQADSPKRLSAAIGKAWEEGVCGIRSATRLENImwkqisnelnhillENDMKFTvvvgdvsGILA
5 DSGCvinwkgrELKCGSGifvtnevHTWTEQYKFQADSPKRLSAAIGKAWEEGVCGIRSATRLENIMwkqisnelnhilleNDMKFTVvvgdvsgILA
6 DSGCVINWKGRELKCGSGIfvtnevhTWTEQYKFQADSPKRLSAAIGKAWEEGVCGIRSATRLENIMWkqisnelnhillendmkftvvVGDVSGILA
7 DSGCVINWKGRELKCGSGIFvtnevhtWTEQYKFQADSPKRLSAAIGKAWEEGVCGIRSATRLENIMWKqisnelnhillendmkftvvvGDVSGILA

1 QGKKMIRPQPMEHKyswkswgkakiigaDVQNTTFIIDGPNTPECPDNQrawniwevedygfgIFTTNIWLKLRDSYTQVCDHRLMSAAIKdskavha
2 QGKKMIRPQPMEHKYswkswgkakiigadVQNTTFIIDGPNTPECPDNQRawniwevEDYGFGIFTTNIWLKLRDSYTQVCDHRLMSAAIKDskavha
3 QGKKMIRPQPMEHKYSwkswgkakiigadvQNTTFIIDGPNTPECPDNQRAwniweveDYGFGIFTTNIWLKLRDSYTQVCDHRLMSAAIKDSkavha
4 QGKKMIRPQPmehkyswKSWGKAKiigadvqNTTFIIDGPNTPECpdnqrawniwevedYGFGIFTTNIWLKLRDSYTQVCDHRLMSAAIKDSKavha
5 QGKKMIRPQPMehkyswkSWGKAKIigadvqnTTFIIDGPNTPECPdnqrawniwevedyGFGIFTTNIWLKLRDSYTQVCDHRLMSAAIKDSKAvha
6 QGKKMIRPQPMEhkyswkswgkakiiGADVQNTTFIIDGPNTPECPDnqrawniwevedygFGIFTTNIWLKLRDSYTQVCDHRLMSAAikdskavha
7 QGKKMIRPQPMEHkyswkswgkakiigADVQNTTFIIDGPNTPECPDNqrawniwevedygfGIFTTNIWLKLRDSYTQVCDHRLMSAAIkdskavha

1 dmgywieSEKNETWKLARASFIEVKTCIWPKSHTLwsngvleSEMIIPKIYGGPISQHNYRPGYFTQTAGpwhlgkleldfdlcegttvvvDEHCGNR
2 dmgywiesEKNETWKLARASFIEVKTCIWPKSHTLWsngvlesEMIIPKIYGGPISQHNYRPGYFTQTAGPwhlgkleldfdlcegttvvvdEHCGNR
3 dmgywieseKNETWKLARASFIEVKTCIWPKSHTLWSngvleseMIIPKIYGGPISQHNYRPGYFTQTAGPWhlgklelDFDLCEGttvvvdeHCGNR
4 dmgywiesekNETWKLARASFIEVKTCIWPKSHTLWSNgvlesemIIPKIYGGPISQHNYRPGYFTQTAGPWHlgkleldFDLCEGTtvvvdehCGNR
5 dmgywieseknETWKLARASFIEVKTCIWPKSHTLWSNGvlesemiIPKIYGGPISQHNYRPGYFTQTAGPWHLgkleldfDLCEGTTvvvdehcGNR
6 dmgywieseknеTWKLARASFIEVKTCIWPKSHTLWSNGVlesemiiPKIYGGPISQHNYRPGYFTQTAGPWHLGkleldfdlcegttvvvdehcgNR
7 dmgywiESEKNETWKLARASFIEVKTCIWPKSHTlwsngvlESEMIIPKIYGGPISQHNYRPGYFTQTAGPWHLGKleldfdlcegttvvVDEHCGNR

1 GPSLRTTtvtgktiHEWCCRSCTLPPLRFKGEDGCwygmeirPVKEKEENLVKSMVSA
2 GPSLRTTTvtgktihEWCCRSCTLPPLRFKGEDGCWygmeirpVKEKEENLVKSMVSA
3 GPSLRTTTVtgktiheWCCRSCTLPPLRFKgedgcwyGMEIRPVKEKEENLVKSMVSA
4 GPSLRTTTVTgktihewCCRSCTLPPLRFKGedgcwygMEIRPVKEKEENLVKSMVSA
5 GPSLRTTTVTGktihewcCRSCTLPPLRFKGEdgcwygmEIRPVKEKEENLVKSMVSA
6 GPSLRTTTVTGKtihewccRSCTLPPLRFKGEDgcwygmeIRPVKEKEENLVKSMVSA
7 GPSLRTTTVTGKTihewccrSCTLPPLRFKGEDGcwygmeiRPVKEKEENLVKSMVSA

DENV2

1 dsgcvvsWKNKELKCGSGIFItdnvhtwTEQYKFQPESPSKLASAIQKAHEEGICGIRSVTRLENLMWKQitpelnhilsenevKLTIMTGDIKGIMQ
2 DsgcvvswKNKELKCGSGIFITdnvhtwtEQYKFQPESPSKLASAIQKAHEEGICGIRSVTRLEnlmwkqitpelnhiLSENEVKLTIMTGDIKGIMQ
3 DSgcvvswkNKELKCGSGIFITDnvhtwteQYKFQPESPSKLASAIQKAHEEGICGIRSVTRLENlmwkqitpelnhilSENEVKLTIMTGDIKGIMQ
4 DSGcvvswknKELKCGSGIFITDNvhtwteqYKFQPESPSKLASAIQKAHEEGICGIRSVTRLENLmwkqitpelnhilsENEVKLTIMTGDIKGIMQ
5 DSGCvvswknkELKCGSGifitdnvHTWTEQYKFQPESPSKLASAIQKAHEEGICGIRSVTRLENLMwkqitpelnhilseNEVKLTIMTGDIKGIMQ
6 DSGCVVSWKNKELKCGSGIfitdnvhTWTEQYKFQPESPSKLASAIQKAHEEGICGIRSVTRLENLMWkqitpelnhilsenEVKLTIMTGDIKGIMQ
7 DSGCVVSWKNKELKCGSGIFitdnvhtWTEQYKFQPESPSKLASAIQKAHEEGICGIRSVTRLENLMWKqitpelnhilseneVKLTIMTGDIKGIMQ

1 AGKRSLRPQPTELKyswktwgkakmlstESHNQTFLIDGPETAECPNTNrawnslevedygfgVFTTNIWLKLREKQdvfcdskLMSAAIKdnravha
2 AGKRSLRPQPTELKYswktwgkakmlsteSHNQTFLIDGPETAECPNTNRawnslevEDYGFGVFTTNIWLKLREKQDvfcdsklMSAAIKDnravha
3 AGKRSLRPQPTELKYSwktwgkakmlstesHNQTFLIDGPETAECPNTNRAwnsleveDYGFGVFTTNIWLKlrekqdvFCDSKLMSAAIKDNravha
4 AGKRSLRPQPtelkyswKTWGKAKmlsteshNQTFLIDGPETAECPNTNRAWnslevedYGFGVFTTNIWLKLrekqdvfCDSKLMSAAIKDNRavha
5 AGKRSLRPQPTelkyswkTWGKAKMlsteshnQTFLIDGPETAECPNTNRAWNslevedyGFGVFTTNIWLKLRekqdvfcDSKLMSAAIKDNRAvha
6 AGKRSLRPQPTElkyswktwgkakmlSTESHNQTFLIDGPETAECPNTNRAWNSlevedygFGVFTTNIWLKLREkqdvfcdSKLMSAAikdnravha
7 AGKRSLRPQPTELkyswktwgkakmlsTESHNQTFLIDGPETAECPNTnrawnslevedygfGVFTTNIWLKLREKqdvfcdsKLMSAAIkdnravha

1 dmgywiesalndtwKMEKASFIEVKSCHWPKSHTLwsngvleSEMIIPKSFAGPVSQHNYRPGYYTQTAGpwhlgklEMDFDFCegttvvvTEDCGNR
2 dmgywiesalndtwkMEKASFIEVKSCHWPKSHTLWsngvlesEMIIPKSFAGPVSQHNYRPGYYTQTAGPwhlgkleMDFDFCEgttvvvtEDCGNR
3 dmgywiesalndtwkmEKASFIEVKSCHWPKSHTLWSngvleseMIIPKSFAGPVSQHNYRPGYYTQTAGPWhlgklemDFDFCEGttvvvteDCGNR
4 dmgywiesalndtwkmeKASFIEVKSCHWPKSHTLWSNgvlesemIIPKSFAGPVSQHNYRPGYYTQTAGPWHlgklemdFDFCEGTtvvvtedCGNR
5 dmgyWIESALNdtwkmekASFIEVKSCHWPKSHTLWSNGvlesemiIPKSFAGPVSQHNYRPGYYTQTAGPWHLgklemdfDFCEGTTvvvtedcGNR
6 dmgywIESALNDtwkmekaSFIEVKSCHWPKSHTLWSNGVlesemiiPKSFAGPVSQHNYRPGYYTQTAGPWHLGklemdfdFCEGTTVvvtedcgNR
7 dmgywiESALNDTwkmekasFIEVKSCHWPKSHTlwsngvlESEMIIPKSFAGPVSQHNYRPGYYTQTAGPWHLGKlemdfdfcegttvvvtedcgnR

1 GPSLRTTtasgkliTEWCCRSCTLPPLRyrgedgcwygmeirPLKEKEENLVNSLVTA
2 GPSLRTTTasgklitEWCCRSCtlpplryRGEDGCWygmeirpLKEKEENLVNSLVTA
3 GPSLRTTTAsgkliteWCCRSCTlpplryrgedgcwyGMEIRPLKEKEENLVNSLVTA
4 GPSLRTTTASgklitewCCRSCTLpplryrgedgcwygMEIRPLKEKEENLVNSLVTA
5 GPSLRTTTASGklitewcCRSCTLPplryrgedgcwygmEIRPLKEKEENLVNSLVTA
6 GPSLRTTTASGKlitewccRSCTLPPlryrgedgcwygmeIRPLKEKEENLVNSLVTA
7 GPSLRTttasgklitewccrSCTLPPLryrgedgcwygmeiRPLKEKEENLVNSLVTA

DENV3

1 dmgcvinWKGKELKcgsgifvtnevhtwTEQYKFQADSPKRLATAIAGAWENGVCGIRSTTRMENLLWKQianelnyilwennikltvvvgDITGVLE
2 DmgcvinwKGKELKCgsgifvtnevhtwtEQYKFQADSPKRLATAIAGAWENGVCGIRSTTRMEnllwkqianelnyiLWENNIKltvvvgdITGVLE
3 DMgcvinwkGKELKCGsgifvtnevhtwteQYKFQADSPKRLATAIAGAWENGVCGIRSTTRMENllwkqianelnyilWENNIKLtvvvgdiTGVLE
4 DMGcvinwkgKELKCGSgifvtnevhtwteqYKFQADSPKRLATAIAGAWENGVCGIRSTTRMENLlwkqianelnyilwENNIKLTvvvgditGVLE
5 DMGCvinwkgkELKCGSGifvtnevHTWTEQYKFQADSPKRLATAIAGAWENGVCGIRSTTRMENLLwkqianelnyilweNNIKLTVvvgditgVLE
6 DMGCVINWKGKELKCGSGIfvtnevhTWTEQYKFQADSPKRLATAIAGAWENGVCGIRSTTRMENLLWkqianelnyilwennikltvvVGDITGVLE
7 DMGCVINWKGKELKCGSGIFvtnevhtWTEQYKFQADSPKRLATAIAGAWENGVCGIRSTTRMENLLWKqianelnyilwennikltvvvGDITGVLE

1 QGKRTLTPQPMELKyswktwgkakivtaETQNSSFIIDGPSTPECPSASrawnvwevedygfgVFTTNIWLKLREVYTQLCDHRlmsaavkderavha
2 QGKRTLTPQPMELKYswktwgkakivtaeTQNSSFIIDGPSTPECPSASRawnvwevEDYGFGVFTTNIWLKLREVYTQLCDHRLmsaavkderavha
3 QGKRTLTPQPMELKYSwktwgkakivtaetQNSSFIIDGPSTPECPSASRAwnvweveDYGFGVFTTNIWLKLREVYTQLCDHRLMsaavkderavha
4 QGKRTLTPQPmelkyswKTWGKAKivtaetqNSSFIIDGPSTPECpsasrawnvwevedYGFGVFTTNIWLKLREVYTQLCDHRLMSaavkderavha
5 QGKRTLTPQPMelkyswkTWGKAKIvtaetqnSSFIIDGPSTPECPsasrawnvwevedyGFGVFTTNIWLKLREVYTQLCDHRLMSAavkderavha
6 QGKRTLTPQPMElkyswktwgkakivTAETQNSSFIIDGPSTPECPSasrawnvwevedygFGVFTTNIWLKLREVYTQLCDHRLMSAAvkderavha
7 QGKRTLTPQPMELkyswktwgkakivtAETQNSSFIIDGPSTPECPSAsrawnvwevedygfGVFTTNIWLKLREVYTQLCDHrlmsaavkderavha

1 dmgywiesqkngswKLEKASLIEVKTCTWPKSHTLwsngvleSDMIIPKSLAGPISQHNHRPGYHTQTAGpwhlgklELDFNYCegttvviSENCGTR
2 dmgywiesqkngswkLEKASLIEVKTCTWPKSHTLWsngvlesDMIIPKSLAGPISQHNHRPGYHTQTAGPwhlgkleLDFNYCEgttvvisENCGTR
3 dmgywiesqkngswklEKASLIEVKTCTWPKSHTLWSngvlesdMIIPKSLAGPISQHNHRPGYHTQTAGPWhlgklelDFNYCEGttvviseNCGTR
4 dmgywiesqkngswkleKASLIEVKTCTWPKSHTLWSNgvlesdmIIPKSLAGPISQHNHRPGYHTQTAGPWHlgkleldFNYCEGTtvvisenCGTR
5 dmgyWIESQKNgswklekASLIEVKTCTWPKSHTLWSNGvlesdmiIPKSLAGPISQHNHRPGYHTQTAGPWHLgkleldfNYCEGTTvvisencGTR
6 dmgywIESQKNGswklekaSLIEVKTCTWPKSHTLWSNGVlesdmiiPKSLAGPISQHNHRPGYHTQTAGPWHLGkleldfnYCEGTTVvisencgTR
7 dmgywiESQKNGSwklekasLIEVKTCTWPKSHTlwsngvlESDMIIPKSLAGPISQHNHRPGYHTQTAGPWHLGKleldfnycegttvvISENCGTR

1 GPSLRTTtvsgkliHEWCCRSCTLPPLRymgedgcwygmeirPINEKEENMVKSLASA
2 GPSLRTTTvsgklihEWCCRSCtlpplryMGEDGCWygmeirpINEKEENMVKSLASA
3 GPSLRTTTVsgkliheWCCRSCTlpplrymgedgcwyGMEIRPINEKEENMVKSLASA
4 GPSLRTTTVSgklihewCCRSCTLpplrymgedgcwygMEIRPINEKEENMVKSLASA
5 GPSLRTTTVSGklihewcCRSCTLPplrymgedgcwygmEIRPINEKEENMVKSLASA
6 GPSLRTTTVSGKlihewccRSCTLPPlrymgedgcwygmeIRPINEKEENMVKSLASA
7 GPSLRTttvsgklihewccrSCTLPPLrymgedgcwygmeiRPINEKEENMVKSLASA

DENV4

1 dmgcvvsWSGKELKCGSGIFVadnvhtwTEQYKFQPESPARLASAILNAHKDGVCGIRSTTRLENVMWKQitnelnyvlwegghdltvvagDVKGVLT
2 DmgcvvswSGKELKCGSGIFVAdnvhtwtEQYKFQPESPARLASAILNAHKDGVCGIRSTTRLEnvmwkqitnelnyvLWEGGHDltvvagdVKGVLT
3 DMgcvvswsGKELKCGSGIFVADnvhtwteQYKFQPESPARLASAILNAHKDGVCGIRSTTRLENvmwkqitnelnyvlWEGGHDLtvvagdvKGVLT
4 DMGcvvswsgKELKCGSGIFVADNvhtwteqYKFQPESPARLASAILNAHKDGVCGIRSTTRLENVmwkqitnelnyvlwEGGHDLTvvagdvkGVLT
5 DMGCvvswsgkELKCGSGifvadnvHTWTEQYKFQPESPARLASAILNAHKDGVCGIRSTTRLENVMwkqitnelnyvlweGGHDLTVvagdvkgVLT
6 DMGCVVSWSGKELKCGSGIfvadnvhTWTEQYKFQPESPARLASAILNAHKDGVCGIRSTTRLENVMWkqitnelnyvlwegghdltvvAGDVKGVLT
7 DMGCVVSWSGKELKCGSGIFvadnvhtWTEQYKFQPESPARLASAILNAHKDGVCGIRSTTRLENVMWKqitnelnyvlwegghdltvvaGDVKGVLT

1 KGKRALTPPVNDLKyswktwgKAKIFTPEARNSTFLIDGPDTSECPNERrawnflevedygfgMFTTNIWMKFREGSSEVCDHRLMSAAIKdqkavha
2 KGKRALTPPVNDLKYswktwgkAKIFTPEARNSTFLIDGPDTSECPNERRawnflevEDYGFGMFTTNIWMKFREGSSEVCDHRLMSAAIKDqkavha
3 KGKRALTPPVNDLKYSwktwgkaKIFTPEARNSTFLIDGPDTSECPNERRAwnfleveDYGFGMFTTNIWMKFREGSSEVCDHRLMSAAIKDQkavha
4 KGKRALTPPVndlkyswKTWGKAKIFTPEARNSTFLIDGPDTSECPNERRAWnflevedYGFGMFTTNIWMKFREGSSEVCDHRLMSAAIKDQKavha
5 KGKRALTPPVNdlkyswkTWGKAKIFTPEARNSTFLIDGPDTSECPNERRAWNflevedyGFGMFTTNIWMKFREGSSEVCDHRLMSAAIKDQKAvha
6 KGKRALTPPVNDlkyswktWGKAKIFTPEARNSTFLIDGPDTSECPNERRAWNFlevedygFGMFTTNIWMKFREGSSEVCDHRLMSAAikdqkavha
7 KGKRALTPPVNDLkyswktwGKAKIFTPEARNSTFLIDGPDTSECPNErrawnflevedygfGMFTTNIWMKFREGSSEVCDHRLMSAAIkdqkavha

1 dmgywiessknqtwQIEKASLIEVKTCLWPKTHTLWSNGVLESQMLIPKSYAGPFSQHNYRQGyatqtvgpwhlgklEIDFGECpgttvtiQEDCDHR
2 dmgywiessknqtwqIEKASLIEVKTCLWPKTHTLWSNGVLESQMLIPKSYAGPFSQHNYRQGYatqtvgpwhlgkleIDFGECPgttvtiqEDCDHR
3 dmgywiessknqtwqiEKASLIEVKTCLWPKTHTLWSNGVLESQMLIPKSYAGPFSQHNYRQGYAtqtvgpwhlgkleiDFGECPGttvtiqeDCDHR
4 dmgywiessknqtwqieKASLIEVKTCLWPKTHTLWSNGVLESQMLIPKSYAGPFSQHNYRQGYATqtvgpwhlgkleidFGECPGTtvtiqedCDHR
5 dmgyWIESSKNqtwqiekASLIEVKTCLWPKTHTLWSNGVLESQMLIPKSYAGPFSQHNYRQGYATQtvgpwhlgkleidfGECPGTTvtiqedcDHR
6 dmgywIESSKNQtwqiekaSLIEVKTCLWPKTHTLWSNGVLESQMLIPKSYAGPFSQHNYRQGYATQTvgpwhlgkleidfgECPGTTVtiqedcdHR
7 dmgywiESSKNQTwqiekasLIEVKTCLWPKTHTLWSNGVLESQMLIPKSYAGPFSQHNYRQgyatqtvGPWHLGKleidfgeCPGTTVTiqedcdhR

1 GPSLRTTtasgklvTQWCCRSCTMPPLRflgedgcwygmeirPLSEKEENMVKSQVTA
2 GPSLRTTTasgklvtQWCCRSCTMPPLRFlgedgcwygmeirpLSEKEENMVKSQVTA
3 GPSLRTTTAsgklvtqWCCRSCTmpplrflgedgcwyGMEIRPLSEKEENMVKSQVTA
4 GPSLRTTTASgklvtqwCCRSCTMpplrflgedgcwygMEIRPLSEKEENMVKSQVTA
5 GPSLRTTTASGklvtqwcCRSCTMPplrflgedgcwygmEIRPLSEKEENMVKSQVTA
6 GPSLRTTTASGKlvtqwccRSCTMPPlrflgedgcwygmeIRPLSEKEENMVKSQVTA
7 GPSLRTTTASGKLvtqwccrSCTMPPLrflgedgcwygmeiRPLSEKEENMVKSQVTA

Appendix S3: peptide libraries of NS1 proteins of DENV serotypes bind to antibody GUS2

DENV1

1 dsgcvinWKGRELKCGSGIFVTNEVHTWteqykfqADSPKRLSAAIGKAWEEGVCGIRSATRLenimwkqISNELNHILLENDMKFTVVVGDVSGILA

2 DsgcvinwKGRELKCGSGIFVTNEVHTWTeqykfqaDSPKRLSAAIGKAWEEGVCGIRSATRLEnimwkqiSNELNHILLENDMKFTVVVGDVSGILA

3 DSgcvinwkGRELKCGSGIFVTNEVHTWTEqykfqadSPKRLSAAIGKAWEEGVCGIRSATRLENimwkqisNELNHILLENDMKFTVVVGDVsgila

4 DSGcvinwkgRELKCGSGIFVTNEvhtwteqykfqadsPKRLSAAIGKAWEEGVCGIRSATRLENImwkqisnELNHILLENDMKFTVVVGDVSgila

5 DSGCvinwkgrELKCGSGIFVTNEVhtwteqyKFQADSPKRLSAAIGKAWEEGVCGIRSAtrlenimWKQISNELNHILLENDMKFTVVVGDVSGila

6 DSGCVinwkgreLKCGSGIFVTNEVHtwteqykFQADSPKRLSAAIGKAWEEGVCGIRSATrlenimwKQISNELNHILLENDMKFTVVVGDVSGIla

7 DSGCVInwkgrelKCGSGIFVTNEVHTwteqykfQADSPKRLSAAIGKAWEEGVCGIRSATRlenimwkQISNELNHILLENDMKFTVVVGDVSGILa

1 qgkkmirpqpmehkyswkswgKAKIIGAdvqnttfIIDGPNTpecpdnqrawniweVEDYGFGIFTTNIWLKLRDSYTQVCDHRLMSAAIKDSKAVHA

2 QgkkmirpqpmehkyswkswgkAKIIGADvqnttfiIDGPNTPecpdnqrawniwevEDYGFGIFTTNIWLKLRDSYTQVCDHRLMSAAIKDSKAVHA

3 qgKKMIRPQpmehkysWKSWGKAkiigadvqnttfiiDGPNTPEcpdnqraWNIWEVEDYGFGIFTTNIWLKLRDSYTQVCDHRLMSAAIKDSKAVHA

4 qgkKMIRPQPmehkyswKSWGKAKiigadvqNTTFIIDGPNTPECpdnqrawNIWEVEDYGFGIFTTNIWLKLRDSYTQVCDHRLMSAAIKDSKAVHA

5 qgkkMIRPQPMehkyswkSWGKAKIigadvqnTTFIIDGPNTPECPdnqrawnIWEVEDYGFGIFTTNIWLKLRDSYTQVCDHRLMSAAIKDSKAvha

6 qgkkmirpqpmehkyswksWGKAKIIgadvqntTFIIDGPNTPECPDnqrawniWEVEDYGFGIFTTNIWLKLRDSYTQVCDHRLMSAAIKDSKAVha

7 qgkkmirpqpmehkyswkswGKAKIIGadvqnttFIIDGPNTPECPDNqrawniwEVEDYGFGIFTTNIWLKLRDSYTQVCDHRLMSAAIKDSKAVHa

1 dmgywieseknetwKLARASFIEVKTCIWPKSHTLWSNGVLESEMIIPKIYGGPISqhnyrpgyftqtagPWHLGKLELDFDLCEGTTVVVDEHCGNR

2 DmgywieseknetwkLARASFIEVKTCIWPKSHTLWSNGVLESEMIIPKIyggpisqhnyrpgyFTQTAGPWHLGKLELDFDLCEGTTVVVDEHCGNR

3 DMgywieseknetwklARASFIEVKTCIWPKSHTLWSNGVLESEMIIPKIYggpisqhnyrpgyfTQTAGPWHLGKLELDFDLCEGTTVVVDEHCGNR

4 DMGywieseknetwklaRASFIEVKTCIWPKSHTLWSNGVLESEMIIPKIYGgpisqhnyrpgyftQTAGPWHLGKLELDFDLCEGTTVVVDEHCGNR

5 dmgywieseknetwklarASFIEVKTCIWPKSHTLWSNGVLESEMIIPKIYGGpisqhnyrpgyftqTAGPWHLGKLELDFDLCEGTTVVVDEHCGNR

6 dmgywieseknetwklaraSFIEVKTCIWPKSHTLWSNGVLESEMIIPKIYGGPisqhnyrpgyftqtAGPWHLGKLELDFDLCEGTTVVVDEHCGNR

7 dmgywieseknetWKLARASFIEVKTCIWPKSHTLWSNGVLESEMIIPKIYGGPIsqhnyrpgyftqtaGPWHLGKLELDFDLCEGTTVVVDEHCGNR

1 GPSLRTTTVTGKTIHEWCCRSctlpplrFKGEDGCWYGMEIRPVKEKEENLVKSMVSA

2 GPSLRTTTVTGKTIHEWCCRSCtlpplrfKGEDGCWYGMEIRPVKEKEENLVKSMVSA

3 GPSLRTTTVTGKTIHEWCCRSCTlpplrfkGEDGCWYGMEIRPVKEKEENLVKSMVSA

4 GPSLRTTTVTGKTIHEWCCRSCTLpplrfkgEDGCWYGMEIRPVKEKEENLVKSMVSA

5 GPSLRTTTVTGKTIHEWCCRSCTLPplrfkgeDGCWYGMEIRPVKEKEENLVKSMVSA

6 GPSLRTTTVTGKTIHEWCCRSCTLPPlrfkgedGCWYGMEIRPVKEKEENLVKSMVSA

7 GPSLRTTTVTGKTIHEWCCRSCTLPPLrfkgedgCWYGMEIRPVKEKEENLVKSMVSA

DENV2

1 dsgcvvswknkelkCGSGIFITDNVHTWteqykfqPESPSKLasaiqkaHEEGICGIRSVTRLenlmwkqITPELNHILSENEVKLTIMTGDIKGIMQ

2 DsgcvvswknkelkcGSGIFITDNVHTWTeqykfqpESPSKLAsaiqkahEEGICGIRSVTRLEnlmwkqiTPELNHILSENEVKLTIMTGDIKGIMQ

3 DSgcvvswkNKELKCGSGIFITDNVHTWTEqykfqpeSPSKLASaiqkaheEGICGIRSVTRLENlmwkqitPELNHILSENEVKLTIMTGDIKGIMQ

4 DSGcvvswknKELKCGSGIFITDNvhtwteqykfqpesPSKLASAiqkaheeGICGIRSVTRLENLmwkqitpELNHILSENEVKLTIMTGDIKGIMQ

5 DSGCvvswknkELKCGSGIFITDNVhtwteqyKFQPESPsklasaiqkaheegICGIRSVtrlenlmWKQITPELNHILSENEVKLTIMTGDIKGIMQ

6 DSGCVvswknkeLKCGSGIFITDNVHtwteqykFQPESPSklasaiqKAHEEGICGIRSVTrlenlmwKQITPELNHILSENEVKLTIMTGDIKGIMQ

7 DSGCVVswknkelKCGSGIFITDNVHTwteqykfQPESPSKlasaiqkAHEEGICGIRSVTRlenlmwkQITPELNHILSENEVKLTIMTGDIKGIMQ

1 AGKRSLRPQPTELKyswktwgKAKMLSTeshnqtfLIDGPETAECPNTNrawnsleVEDYGFGVFTTNIWLKLREKQDVFCDSKLMSAAIKDNRAVHA

2 AGKRSLRPQPTELKYswktwgkAKMLSTEshnqtflIDGPETAECPNTNRawnslevEDYGFGVFTTNIWLKLREKQDVFCDSKLMSAAIKDNRAVHA

3 AGKRSLRPQptelkysWKTWGKAkmlstesHNQTFLIDGPETAEcpntnraWNSLEVEDYGFGVFTTNIWLKLREKQDVFCDSKLMSAAIKDNRAVHA

4 AGKRSLRPQPtelkyswKTWGKAKmlsteshNQTFLIDGPETAECpntnrawNSLEVEDYGFGVFTTNIWLKLREKQDVFCDSKLMSAAIKDNRAVHA

5 AGKRSLRPQPTelkyswkTWGKAKMlsteshnQTFLIDGPETAECPntnrawnSLEVEDYGFGVFTTNIWLKLREKQDVFCDSKLMSAAIKDNRAvha

6 AGKRSLRPQPTElkyswktWGKAKMLsteshnqTFLIDGPETAECPNtnrawnsLEVEDYGFGVFTTNIWLKLREKQDVFCDSKLMSAAIKDNRAVha

7 AGKRSLRPQPTELkyswktwGKAKMLSteshnqtFLIDGPETAECPNTnrawnslEVEDYGFGVFTTNIWLKLREKQDVFCDSKLMSAAIKDNRAVHa

1 dmgywiesalndtwKMEKASFIEVKSCHWPKSHTLWSNGVLESEMIIPKSFAGPVSqhnyrpgyytqtagPWHLGKLEMDFDFCEGTTVVVTEDCGNR

2 DmgywiesalndtwkMEKASFIEVKSCHWPKSHTLWSNGVLESEMIIPKSfagpvsqhnyrpgyYTQTAGPWHLGKLEMDFDFCEGTTVVVTEDCGNR

3 DMgywiesalndtwkmEKASFIEVKSCHWPKSHTLWSNGVLESEMIIPKSFagpvsqhnyrpgyyTQTAGPWHLGKLEMDFDFCEGTTVVVTEDCGNR

4 DMGywiesalndtwkmeKASFIEVKSCHWPKSHTLWSNGVLESEMIIPKSFAgpvsqhnyrpgyytQTAGPWHLGKLEMDFDFCEGTTVVVTEDCGNR

5 dmgyWIESALNdtwkmekASFIEVKSCHWPKSHTLWSNGVLESEMIIPKSFAGpvsqhnyrpgyytqTAGPWHLGKLEMDFDFCEGTTVVVTEDCGNR

6 dmgywIESALNDtwkmekaSFIEVKSCHWPKSHTLWSNGVLESEMIIPKSFAGPvsqhnyrpgyytqtAGPWHLGKLEMDFDFCEGTTVVVTEDCGNR

7 dmgywiesalndtWKMEKASFIEVKSCHWPKSHTLWSNGVLESEMIIPKSFAGPVsqhnyrpgyytqtaGPWHLGKLEMDFDFCEGTTVVVTEDCGNR

1 GPSLRTTTASGKLITEWCCRSctlpplryrgedgcWYGMEIRPLKEKEENLVNSLVTA

2 GPSLRTTTASGKLITEWCCRSCtlpplryRGEDGCWYGMEIRPLKEKEENLVNSLVTA

3 GPSLRTTTASGKLITEWCCRSCTlpplryrGEDGCWYGMEIRPLKEKEENLVNSLVTA

4 GPSLRTTTASGKLITEWCCRSCTLpplryrgEDGCWYGMEIRPLKEKEENLVNSLVTA

5 GPSLRTTTASGKLITEWCCRSCTLPplryrgeDGCWYGMEIRPLKEKEENLVNSLVTA

6 GPSLRTTTASGKLITEWCCRSCTLPPlryrgedGCWYGMEIRPLKEKEENLVNSLVTA

7 GPSLRTTTASGKLITEWCCRSCTLPPLryrgedgCWYGMEIRPLKEKEENLVNSLVTA

DENV3

1 dmgcvinWKGKELKCGSGIFVTNEVHTWteqykfqADSPKRLATAIAGAWENGVCGIRSTTRMenllwkqIANELNYilwenniKLTVVVGDITGVLE

2 DmgcvinwKGKELKCGSGIFVTNEVHTWTeqykfqaDSPKRLATAIAGAWENGVCGIRSTTRMEnllwkqiANELNYIlwennikLTVVVGDITGVLE

3 DMgcvinwkGKELKCGSGIFVTNEVHTWTEqykfqadSPKRLATAIAGAWENGVCGIRSTTRMENllwkqiaNELNYILwenniklTVVVGDITGVLE

4 DMGcvinwkgKELKCGSGIFVTNEvhtwteqykfqadsPKRLATAIAGAWENGVCGIRSTTRMENLlwkqianELNYILWennikltVVVGDITGVLE

5 DMGCvinwkgkELKCGSGIFVTNEVhtwteqyKFQADSPKRLATAIAGAWENGVCGIRSTtrmenllWKQIANElnyilweNNIKLTVVVGDITGVLE

6 DMGCVinwkgkeLKCGSGIFVTNEVHtwteqykFQADSPKRLATAIAGAWENGVCGIRSTTrmenllwKQIANELnyilwenNIKLTVVVGDITGVLE

7 DMGCVInwkgkelKCGSGIFVTNEVHTwteqykfQADSPKRLATAIAGAWENGVCGIRSTTRmenllwkQIANELNyilwennIKLTVVVGDITGVLE

1 QGKRTLTPQPMELKyswktwgKAKIVTAetqnssfIIDGPSTPECPSASrawnvweVEDYGFGVFTTNIWLKLREVYTQLCDHRLMSAAVKDERAVHA

2 QGKRTLTPQPMELKYswktwgkAKIVTAEtqnssfiIDGPSTPECPSASRawnvwevEDYGFGVFTTNIWLKLREVYTQLCDHRLMSAAVKDERAVHA

3 QGKRTLTPQpmelkysWKTWGKAkivtaetqnssfiiDGPSTPEcpsasraWNVWEVEDYGFGVFTTNIWLKLREVYTQLCDHRLMSAAVKDERAVHA

4 QGKRTLTPQPmelkyswKTWGKAKivtaetqNSSFIIDGPSTPECpsasrawNVWEVEDYGFGVFTTNIWLKLREVYTQLCDHRLMSAAVKDERAVHA

5 QGKRTLTPQPMelkyswkTWGKAKIvtaetqnSSFIIDGPSTPECPsasrawnVWEVEDYGFGVFTTNIWLKLREVYTQLCDHRLMSAAVKDERAvha

6 QGKRTLTPQPMElkyswktWGKAKIVtaetqnsSFIIDGPSTPECPSasrawnvWEVEDYGFGVFTTNIWLKLREVYTQLCDHRLMSAAVKDERAVha

7 QGKRTLTPQPMELkyswktwGKAKIVTaetqnssFIIDGPSTPECPSAsrawnvwEVEDYGFGVFTTNIWLKLREVYTQLCDHRLMSAAVKDERAVHa

1 dmgywiesqkngswKLEKASLIEVKTCTWPKSHTLWSNGVLESDMIIPKSLAGPISqhnhrpgyhtqtagPWHLGKLeldfnycEGTTVVISENCGTR

2 DmgywiesqkngswkLEKASLIEVKTCTWPKSHTLWSNGVLESDMIIPKSlagpisqhnhrpgyHTQTAGPWHLGKLEldfnyceGTTVVISENCGTR

3 DMgywiesqkngswklEKASLIEVKTCTWPKSHTLWSNGVLESDMIIPKSLagpisqhnhrpgyhTQTAGPWHLGKLELdfnycegTTVVISENCGTR

4 DMGywiesqkngswkleKASLIEVKTCTWPKSHTLWSNGVLESDMIIPKSLAgpisqhnhrpgyhtQTAGPWHlgkleldFNYCEGTTVVISENCGTR

5 dmgywiesqkngswklekASLIEVKTCTWPKSHTLWSNGVLESDMIIPKSLAGpisqhnhrpgyhtqTAGPWHLgkleldfNYCEGTTVVISENCGTR

6 dmgywiesqkngswklekaSLIEVKTCTWPKSHTLWSNGVLESDMIIPKSLAGPisqhnhrpgyhtqtAGPWHLGkleldfnYCEGTTVVISENCGTR

7 dmgywiesqkngsWKLEKASLIEVKTCTWPKSHTLWSNGVLESDMIIPKSLAGPIsqhnhrpgyhtqtaGPWHLGKleldfnyCEGTTVVISENCGTR

1 GPSLRTTTVSGKLIHEWCCRSctlpplrYMGEDGCWYGMEIRPINEKEENMVKSLASA

2 GPSLRTTTVSGKLIHEWCCRSCtlpplryMGEDGCWYGMEIRPINEKEENMVKSLASA

3 GPSLRTTTVSGKLIHEWCCRSCTlpplrymGEDGCWYGMEIRPINEKEENMVKSLASA

4 GPSLRTTTVSGKLIHEWCCRSCTLpplrymgEDGCWYGMEIRPINEKEENMVKSLASA

5 GPSLRTTTVSGKLIHEWCCRSCTLPplrymgeDGCWYGMEIRPINEKEENMVKSLASA

6 GPSLRTTTVSGKLIHEWCCRSCTLPPlrymgedGCWYGMEIRPINEKEENMVKSLASA

7 GPSLRTTTVSGKLIHEWCCRSCTLPPLrymgedgCWYGMEIRPINEKEENMVKSLASA

DENV4

1 DMGCVVSWSGKELKCGSGIFVADNVHTWteqykfqPESPARLasailnaHKDGVCGIRSTTRLenvmwkqITNELNYVLWEGGHDLTVVAGDVKGVLT
2 DMGCVVSWSGKELKCGSGIFVADNVHTWTeqykfqpESPARLAsailnahKDGVCGIRSTTRLEnvmwkqiTNELNYVLWEGGHDLTVVAGDVKGVLT
3 DMGCVVSWSGKELKCGSGIFVADNVHTWTEqykfqpeSPARLASailnahkDGVCGIRSTTRLENvmwkqitNELNYVLWEGGHDLTVVAGDVKGVLT
4 DMGCVVSWSGKELKCGSGIFVADNvhtwteqykfqpesPARLASAilnahkdGVCGIRSTTRLENVmwkqitnELNYVLWEGGHDLTVVAGDVKGVLT
5 DMGCVVSWSGKELKCGSGIFVADNVhtwteqyKFQPESPARLASAIlnahkdgVCGIRSTtrlenvmWKQITNELNYVLWEGGHDLTVVAGDVKGVLT
6 DMGCVVSWSGKELKCGSGIFVADNVHtwteqykFQPESPARLASAILnahkdgvCGIRSTTrlenvmwKQITNELNYVLWEGGHDLTVVAGDVKGVLT
7 DMGCVVSWSGKELKCGSGIFVADNVHTwteqykfQPESPARLASAILNahkdgvcGIRSTTRlenvmwkQITNELNYVLWEGGHDLTVVAGDVKGVLT

1 KGKRALTPPVNDLKyswktwgKAKIFTPearnstfLIDGPDTsecpnerrawnfleVEDYGFGMFTTNIWMKFREGSSEVCDHRLMSAAIKdqkavha
2 KGKRALTPPVNDLKYswktwgkAKIFTPEarnstflIDGPDTSecpnerrawnflevEDYGFGMFTTNIWMKFREGSSEVCDHRLmsaaikdqkavha
3 KGKRALTPPvndlkysWKTWGKAkiftpearnstfliDGPDTSEcpnerraWNFLEVEDYGFGMFTTNIWMKFREGSSEVCDHRLMsaaikdqKAVHA
4 KGKRALTPPVndlkyswKTWGKAKiftpearNSTFLIDGPDTSECpnerrawNFLEVEDYGFGMFTTNIWMKFREGSSEVCDHRLMSaaikdqkAVHA
5 KGKRALTPPVNdlkyswkTWGKAKIftpearnSTFLIDGPDTSECPnerrawnFLEVEDYGFGMFTTNIWMKFREGSSEVCDHRLMSAaikdqkavha
6 KGKRALTPPVNDlkyswktWGKAKIFtpearnsTFLIDGPDTSECPNerrawnfLEVEDYGFGMFTTNIWMKFREGSSEVCDHRLMSAAikdqkavha
7 KGKRALTPPVNDLkyswktwGKAKIFTpearnstFLIDGPDTSECPNErrawnflEVEDYGFGMFTTNIWMKFREGSSEVCDHRLMSAAIkdqkavha

1 dmgywiessknqtwQIEKASLIEVKTCLWPKTHTLWSNGVLESQMLIPKSYAGPFSqhnyrqgYATQTVGPWHLGKLEIDFGECPGTTVTIQEDCDHR
2 dmgywiessknqtwqIEKASLIEVKTCLWPKTHTLWSNGVLESQMLIPKSyagpfsqhnyrqgyATQTVGPWHLGKLEIDFGECPGTTVTIQEDCDHR
3 DMgywiessKNQTWQIEKASLIEVKTCLWPKTHTLWSNGVLESQMLIPKSYagpfsqhnyrqgyaTQTVGPWHLGKLEIDFGECPGTTVTIQEDCDHR
4 DMGywiesskNQTWQIEKASLIEVKTCLWPKTHTLWSNGVLESQMLIPKSYAgpfsqhnyrqgyatQTVGPWHLGKLEIDFGECPGTTVTIQEDCDHR
5 dmgywiessknQTWQIEKASLIEVKTCLWPKTHTLWSNGVLESQMLIPKSYAGpfsqhnyrqgyatqTVGPWHLGKLEIDFGECPGTTVTIQEDCDHR
6 dmgywiessknqTWQIEKASLIEVKTCLWPKTHTLWSNGVLESQMLIPKSYAGPfsqhnyrQGYATQTVGPWHLGKLEIDFGECPGTTVTIQEDCDHR
7 dmgywiessknqtWQIEKASLIEVKTCLWPKTHTLWSNGVLESQMLIPKSYAGPFsqhnyrqGYATQTVGPWHLGKLEIDFGECPGTTVTIQEDCDHR

1 GPSLRTTTASGKLVtqwccrsctmpplrFLGEDGCWYGMEIRPLSEKEENMVKSQVTA
2 GPSLRTTTASGKLVTqwccrsctmpplrfLGEDGCWYGMEIRPLSEKEENMVKSQVTA
3 GPSLRTTTAsgklvtqWCCRSCTmpplrflGEDGCWYGMEIRPLSEKEENMVKSQVTA
4 GPSLRTTTASgklvtqwCCRSCTMpplrflgEDGCWYGMEIRPLSEKEENMVKSQVTA
5 GPSLRTTTASGklvtqwcCRSCTMPplrflgeDGCWYGMEIRPLSEKEENMVKSQVTA
6 GPSLRTTTASGKlvtqwccRSCTMPPlrflgedGCWYGMEIRPLSEKEENMVKSQVTA
7 GPSLRTTTASGKLvtqwccrSCTMPPLrflgedgCWYGMEIRPLSEKEENMVKSQVTA
